# Experimental, numerical, and data-driven analysis of impulsive ice-shedding-induced vibrations in wind turbine blades

**DOI:** 10.1038/s41598-026-53564-7

**Published:** 2026-05-28

**Authors:** Iyad F. Al-Najjar, Károly Jálics, László E. Kollár

**Affiliations:** 1 Department of Mechanical and Industrial Engineering, Applied Science Private University, Amman, Jordan; 2 Institute of Machine and Product Design, University of Miskolc, Miskolc, Hungary; 3 Savaria Institute of Technology, ELTE Eötvös Loránd University, Budapest, Hungary

**Keywords:** Energy science and technology, Engineering, Mathematics and computing

## Abstract

This research investigates the dynamic response of a glass-filled nylon wind turbine blade subjected to ice shedding and turbulent wind conditions, with the aim of understanding and predicting structural behaviour under realistic operating scenarios. Experiments were conducted to capture the blade’s vibration response under controlled conditions, with the blade fixed at the root and measurements taken at the mid-span and tip. Ice shedding was modelled by attaching masses ranging from 100 g to 1 kg at various positions and release angles (− 5°, 0°, 5°, and 15°), allowing evaluation of how sudden mass loss influences dynamic behaviour; the first peak response was analysed as it represents the maximum deflection and highest risk of structural failure. A finite element model was developed and validated using the experimental results to accurately represent the blade’s structural dynamics and predict its natural frequencies and mode shapes, with frequency differences below 0.6% confirming model accuracy. Random vibration analysis using the Kaimal turbulence spectrum with frequency differences below 0.6% confirming model accuracy. In addition, neural network models such as Multi-Layer Perceptron, Levenberg-Marquardt (LM), and Scaled Conjugate Gradient were trained on 140 data points to predict trends in blade displacement amplitude as a function of ice mass, sensor location, and blade orientation under ice shedding and sudden mass loss scenarios, with Bayesian Regularization achieving the best performance (RMSE = 0.0032). While the blade response follows classical bending behaviour, the novelty of this study lies in the experimental methodology and integrated framework for evaluating ice-shedding effects. A noticeable change in the response is observed when the released mass becomes comparable to the blade mass, demonstrating the sensitivity of the method to mass variation. Overall, the approach provides a basis for design evaluation and structural monitoring of wind turbine blades under ice shedding and turbulent wind conditions.

## Introduction

Wind turbine blades operating in cold and humid environments are exposed to atmospheric icing, which can significantly affect both aerodynamic performance and structural integrity. Ice accretion on blades alters mass distribution, stiffness, and damping characteristics, and can lead to imbalance, increased vibration levels, and elevated structural loads^1^. Wind-induced vibration and structural loading remain important challenges in wind turbine systems, where dynamic response and vibration mitigation strategies continue to receive significant research attention under complex environmental loading conditions^2,3^. Of particular concern is ice shedding, which occurs when accumulated ice detaches from the blade during operation or shutdown. Ice shedding introduces impulsive excitation to the structure and may result in transient vibrations that can affect fatigue life, serviceability, and, in extreme cases, structural safety.

While extensive research has focused on aerodynamic losses and power reduction due to blade icing, the structural dynamic response of blades during ice shedding has received comparatively less attention^4,5^. Similar transient vibration behaviour has also been investigated in beam-like structures subjected to moving and attached masses, where mass interaction significantly influenced the dynamic response characteristics^6^. For wind turbine blades, a relatively small ice mass or wind gusts can generate noticeable transient responses due to their low mass and stiffness^7^. For instance, a bird impact of an operating blade can cause such problems^8^. Beyond aerodynamic effects, uneven ice accretion and sudden ice release can induce significant vibration and transient imbalance in blade structures, amplifying localized stresses and contributing to fatigue damage. Reviews of icing phenomena have highlighted that ice shedding may occur as a result of structural vibrations and bending deformations of blades and that this shedding itself introduces dynamic loading beyond steady aerodynamic losses^9^, Furthermore, studies specifically examining icing effects on wind turbine structural dynamics show that blade icing can increase vibration under combined environmental loads, with dynamic responses that vary with operating conditions and blade loading regimes^10^. Detailed modal analyses have also demonstrated that ice accretion alters the modal properties and vibration behaviour of blades, such as changes in natural frequencies and damping that can worsen structural responses under operational loads^11^. Additionally, advanced aeroelastic studies incorporating geometric nonlinearities reveal how icing mass distribution and nonuniform flow conditions significantly impact dynamic blade response, underscoring the need to include structural dynamics in ice-related assessments^12^. Understanding these ice-induced structural vibrations and transient dynamic effects is therefore essential for improving blade design, predictive modelling, condition monitoring, and operational safety in cold and humid environments. The experimental part of this study aims to develop a methodology for conducting modal analysis experiment on wind turbine blades under ice-shedding conditions, by using an attached mass and examining the resulting vibrations following sudden mass (ice) release.

### Experimental study

Wind turbine blades are fixed at the root to the tower, while being free at the tip. Hence, they can be treated as cantilever beams in both experimental and numerical analysis^13,14^. Work. Experimental modal analysis and vibration testing of cantilever-like structures have been widely used to identify natural frequencies, mode shapes, and damping characteristics under controlled conditions^15^. These approaches provide a fundamental basis for validating numerical models and for interpreting complex dynamic responses.

Several experimental studies have investigated the free vibration response of cantilever beams and blade-like structures subjected to impulsive or transient loading^16^, which focus on the maximum amplitude under different aspect ratios. In another study^17^ the coupled bending–bending vibration of pre-twisted tapered cantilever blades was analysed, demonstrating that taper significantly influences vibration frequencies and modal shapes, with theoretical predictions closely matching experimental results.

In another study, down-scaled wind turbine blades underwent modal, static, and fatigue testing to validate structural models^18^, while mild steel cantilever beams with and without end masses were analysed experimentally, numerically, and analytically^19^. Results showed close agreement between experiments and simulations, with deviations in analytical predictions due to neglected mass effects. The findings highlight the importance of accurate modelling, including damping, mass, and boundary conditions, for predicting modal behaviour, resonant frequencies, and structural reliability.

Such findings are directly relevant to ice-shedding events, where sudden mass release introduces an impulsive excitation that drives the structure into an underdamped free-vibration response. However, it is still missing an actual modelling of ice shedding and an analysis for the maximum amplitude of the free vibration for wind turbine blades after the release, which this research aims to investigate.

The main contribution of the present work is the development of an experimental framework for reproducing impulsive ice-shedding events through controlled mass attachment and rapid release on a small-scale wind turbine blade. This setup provides a starting point for investigating the transient vibration behaviour of small-scale blade models under simulated ice-shedding conditions. While finite element and neural network analyses are included as supporting tools for validation and interpretation, the primary objective is to establish a repeatable experimental methodology that can be extended in future studies to larger wind turbine blade systems.

### Numerical study

In parallel with experimental work, different numerical methods are used to study wind turbine blades such as the Method of lines in^20^ or finite element modelling to predict modal properties and dynamic response of beam-like structures or prediction mass loads over a wind turbine blade^21^. Validation of FEM models using experimentally measured natural frequencies is commonly performed using error metrics such as root mean square (RMS) error, especially when material properties such as Young’s modulus are uncertain^22^. These validated models form the foundation for more advanced analyses, including random vibration and probabilistic response assessment^23^, which studied the structural response under stochastic wind excitation, where loading is described statistically using power spectral density (PSD) functions rather than time histories. While this approach does not capture extreme transient events, it provides valuable insight into root mean square (RMS) displacement, acceleration, and stress levels under turbulent wind conditions. When combined with experimental validation and modal-based modelling, random vibration analysis offers an efficient framework for assessing the dynamic behaviour of lightweight blades subjected to realistic operating environments.

Random vibration analysis has been widely used for analysing the dynamic response of wind turbine blades or slender structural components subjected to stochastic wind excitation. Several studies have demonstrated that turbulent wind loading can be effectively represented in the frequency domain using power spectral density formulations^24^, allowing efficient evaluation of structural response without resorting to time-domain simulations^25^.

The Von Kármán turbulence spectrum is commonly employed to describe atmospheric turbulence due to its realistic representation of energy distribution across frequencies^26^. Its suitability for wind-induced vibration analysis has been highlighted in studies focusing on wind turbine structures, where low-frequency turbulence components were shown to dominate the excitation of bending modes^27^. These findings support the use of frequency-domain methods for identifying modal contributions and estimating RMS response quantities.

Due to the lack of real wind records to perform dynamic analysis of structural systems, structural engineers commonly use simplified and conservative approaches to consider the dynamic effects of wind^28^. Furthermore, several investigations have applied finite element–based random vibration analysis to beam-like structures under stochastic excitation. In these studies, the governing equations of motion are transformed into the frequency domain, and the response PSD is obtained through frequency response functions (FRFs). The resulting RMS values of displacement, velocity, or stress are then used as indicators of vibration severity rather than deterministic response histories^29^.

Random vibration approaches have also been applied to wind turbine blades to evaluate deformation, acceleration, and stress distributions under broadband excitation by the use of different methods such as machine learning^30^, or using sensors as accelerometers in real life^31^. Another study^32^, which highlights that such analyses provide statistical measures of response, which are particularly useful for comparative studies, preliminary design assessment, and sensitivity analysis, but are not intended to replace detailed aeroelastic time-domain simulations.

Numerical simulations of turbulent wind are used to compute stochastic aerodynamic loads on Vertical Axis Wind Turbines (VAWTs), enabling evaluation of blade stresses and fatigue beyond steady-wind assumptions^33^.

The study investigates vibration mitigation of monopile offshore wind turbines using a novel Extended KDamper system, combining stochastic wind and sea wave loads with structural modelling to enhance damping, reliability, and lifespan^34^.

Wind turbine blades operating in atmospheric conditions are subjected to turbulent flow, which is commonly modelled using spectral descriptions such as the Von Kármán power spectral density (PSD), producing broadband stochastic aerodynamic excitation that induces structural vibrations^35,36^. In addition to this, flow separation and wake interactions can generate vortex shedding, resulting in both periodic and random aerodynamic forces that may interact with the blade’s natural frequencies and increase the dynamic response^37^. Under icing conditions, the accumulation of ice changes both the aerodynamic characteristics and the mass distribution of the blade, which significantly affects its dynamic behaviour. It has been reported that structural vibration and bending can contribute to ice detachment, meaning that ice shedding can be triggered or influenced by aerodynamic excitation^38,39^. Therefore, ice shedding can be treated as a transient event caused by a sudden change in mass and superimposed on the stochastic response induced by turbulence. In this study, experimental mass release tests are used to simulate the ice-shedding event and to capture the corresponding peak response. The finite element model is validated using modal properties obtained from these experiments, and the validated model is then used to evaluate the stochastic response under turbulent wind excitation using a Von Kármán-based PSD. This approach links turbulence-induced vibration with ice-shedding-induced transient response within a single framework.

### Neural network

In the literature related to modal analysis (vibration) and neural networks, the focus is to monitor the blade^40^ and to predict cracks^41^. Furthermore, neural networks are increasingly applied in modal analysis of wind turbine blades^42^ to address the complexity and nonlinearity inherent in their dynamic behaviour. By learning patterns directly from measured or simulated vibration data, neural networks enable efficient identification of natural frequencies, mode shapes, and damping characteristics without relying solely on simplified physical models. This data-driven approach improves the accuracy of structural health monitoring and supports more reliable assessment of blade integrity under varying operational and environmental conditions.

Previous work has demonstrated that added mass can be estimated from shifts in natural frequencies using artificial neural networks, with validation through finite element modelling and experimental modal analysis^43^. The results indicate that prediction accuracy improves for larger ice masses, highlighting the potential of this approach for preliminary ice accretion assessment on wind turbine blades^44^. Another AI-based approach has been proposed to identify delamination’s in laminated composite structures by analysing modal behaviour^45^. Experimental and numerical modal data revealed that damage caused measurable reductions in natural frequencies and changes in mode shapes, and a neural network trained on these data was able to predict delamination size and location with high accuracy. Previous works has also demonstrated that neural networks can be used to predict the effects of preload magnitude and position on the natural frequencies of prestressed cantilever beams, reducing computational effort compared to full finite element simulations^46^. A dataset generated from a simple FEM model was used to train the network, and the resulting predictions for different beam geometries and preload conditions showed good agreement with FEM results, highlighting the efficiency of NN-based approximations for structural analysis. Using similar neural network–based strategies, it has also been demonstrated that the dynamic response of nonlinear structures, such as simplified wind turbine models, can be accurately predicted from finite element simulation data^47^. A single-layer network was able to replicate the structural response while reducing computational time by nearly two orders of magnitude, highlighting the potential of neural networks to efficiently approximate complex structural behaviour beyond linear analyses.

This research aims to use neural networks to build on prior works using to predict structural response (maximum amplitude) from modal experiments data which remains unexplored. The prediction of these peak responses could fill a critical gap in wind turbine structural health monitoring and operational safety.

**Fig. 1:**
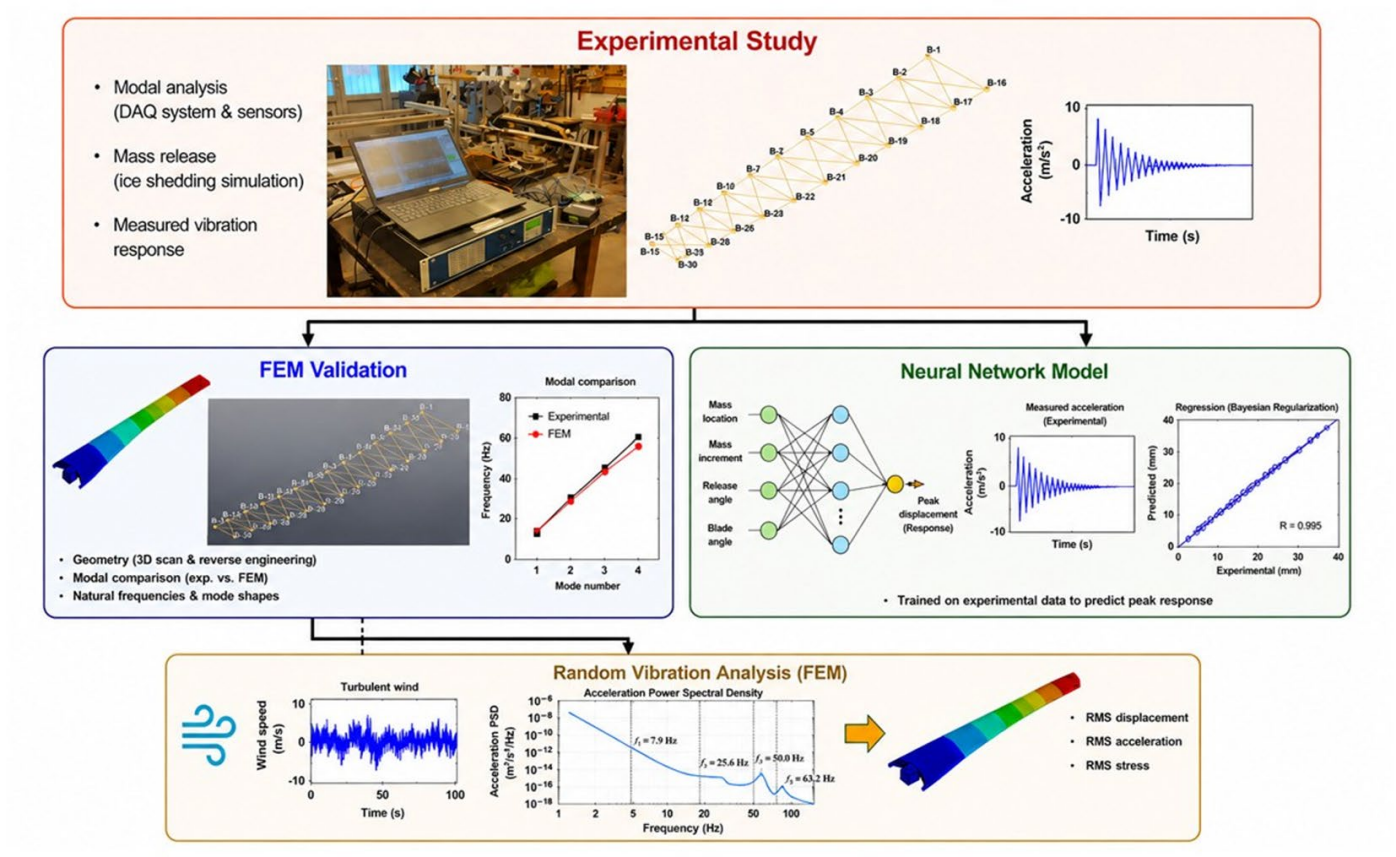
Overall framework of the study showing experimental measurements, FEM validation, neural network prediction, and random vibration analysis under turbulent wind excitation.

Figure 1 illustrates the overall framework of the study, showing the integration of experimental, numerical, and data-driven approaches for analysing wind turbine blade vibrations under ice-shedding and turbulent wind conditions. Experimental data are used to validate the finite element model through comparison of natural frequencies and mode shapes, while the maximum displacement values are used to train the neural network. The validated FEM model is subsequently employed for random vibration analysis using a turbulence-based power spectral density (PSD) input to estimate RMS displacement, acceleration, and stress under stochastic wind excitation.

## Methodology

This research focused on conducting experimental modal analysis on a wind turbine blade manufactured from glass-filled nylon. In the experimental setup, the blade was clamped at the root, while a shaker was used to excite the structure in order to determine its natural frequencies. In a second set of experiments, a mass was attached either to the free end (tip) or to the middle section of the blade and subsequently released, while the dynamic response of the blade was measured. Different values of attached mass and different angles of the fixed support were considered, as described later in this section.

Under real operating conditions and cold environments, ice can accrete on wind turbine blades and may eventually slip or shed from the surface. The attached mass in these experiments was therefore used to model the effect of ice accretion and shedding. In total, eighty different configurations were investigated, with measurements taken at both the mid-span and the free end, resulting in 140 acceleration signals. These experimental results were then used to train and evaluate different neural network algorithms in MATLAB, including Multi-Layer Perceptron (MLP), and Scaled Conjugate Gradient MLP (MLP-SCG), in order to assess the feasibility (evaluate applicability) of applying such techniques to wind turbine blades under ice slipping conditions (ice-release scenarios).

### Experimental modal analysis of a small-scale wind turbine blade model

Experimental modal analysis is conducted to study the dynamic characteristics of a structure in the frequency domain. This is achieved by placing accelerometers at different locations on the structure and exciting it is using a controlled excitation source, such as a shaker or an impact hammer. After mapping the structure with sensors and importing the measured frequency response (FRF) data into a computer for post-processing, the natural frequencies and mode shapes of the structure can be extracted.

Two sets of experiments were conducted in this study. The first one investigated the natural frequencies of the blade while the second one investigated the dynamic response of the blade after releasing an attached mass simulating the process of ice-shedding. In these experiments, the data acquisition was a Brüel & Kjær device, while the RT Pro 7.41 software was used to process the measured data. Figure 1 illustrate the blade geometry and the sensor locations used to conduct the modal experiment and to determine the natural frequencies of the blade.

**Fig. 2:**
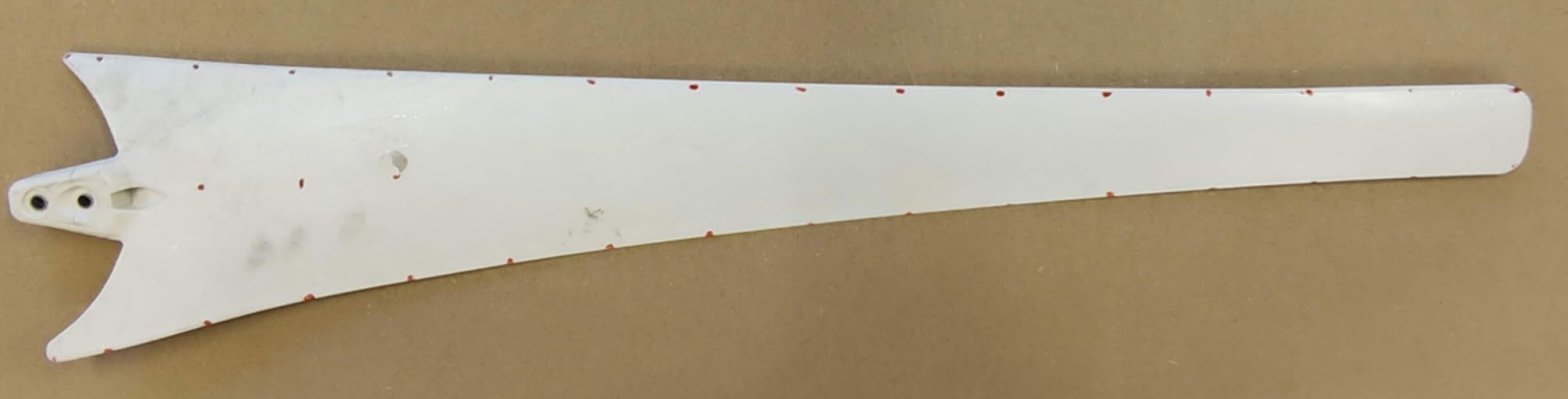
Sensor placement.

#### Modal experiment with attaching and releasing a mass

Further experiments were conducted on the blade, as shown in Fig. 2. The angle of the fixture, θ (support angle, used to approximate the gravitational component of the azimuthal position of a real turbine blade), can be adjusted, and the angles considered in this study were {−5°, 0°, 5°, 15°}. The attached masses ranged from 100 g to 1 kg, increasing in 100 g increments. Each mass was suspended (hung) from the blade using a rope and then released (detached) using a lighter by burning the rope, ensuring that the release was rapid and nearly instantaneous. This method was found to be more effective (more reliable) compared to attaching the mass using a copper wire and cutting it, as reported in previous research^48^.

The selected mass range was chosen to correspond to realistic ice accumulation conditions for the scale of the experimental blade, based on the review by^49^. The lower end of the range (100–300 g) represents light icing, such as soft rime forming during turbine operation, while the higher end (500 g–1 kg) simulates more extreme accumulation under snow or idle operation, where ice may persist and grow over extended periods. The review reports that ice accretion on large wind turbine blades can reach up to 0.8 m in thickness at the leading edge after four days of heavy snowing, with soft rime (~ 350 kg/m³) forming gradually during normal icing and harder rime (~ 550 kg/m³) developing underneath after prolonged exposure. Ice was concentrated at the leading edge and tip to replicate typical field patterns observed in wind turbine blades. This approach ensures that the experiments capture the most relevant transient dynamic effects associated with ice shedding, supporting the physical representativeness and validity of the experimental methodology, as also discussed in our previous simulation study^21^.

Furthermore, Fig. 2 illustrates how the mass was attached to the blade. Rather than tying the rope directly around the blade, the rope was tied around a copper wire to form a knot, which held the mass in place. This arrangement minimizes or eliminates the generation of twisting moments and ensures that the applied load acts primarily as a transverse force (bending load). To minimize any swinging motion effects on the measured response, the mass was positioned less than five centimetres away from the blade in all experiments.

Additionally, after the mass reached a rest state (static equilibrium) and no oscillation was visibly detectable, a settling time of at least one minute was implemented to ensure that all remaining motion had dissipated prior to measurement, thereby minimizing the measured noise. Furthermore, two accelerometers were placed at the tip and the middle part of the blade.

**Fig. 3:**
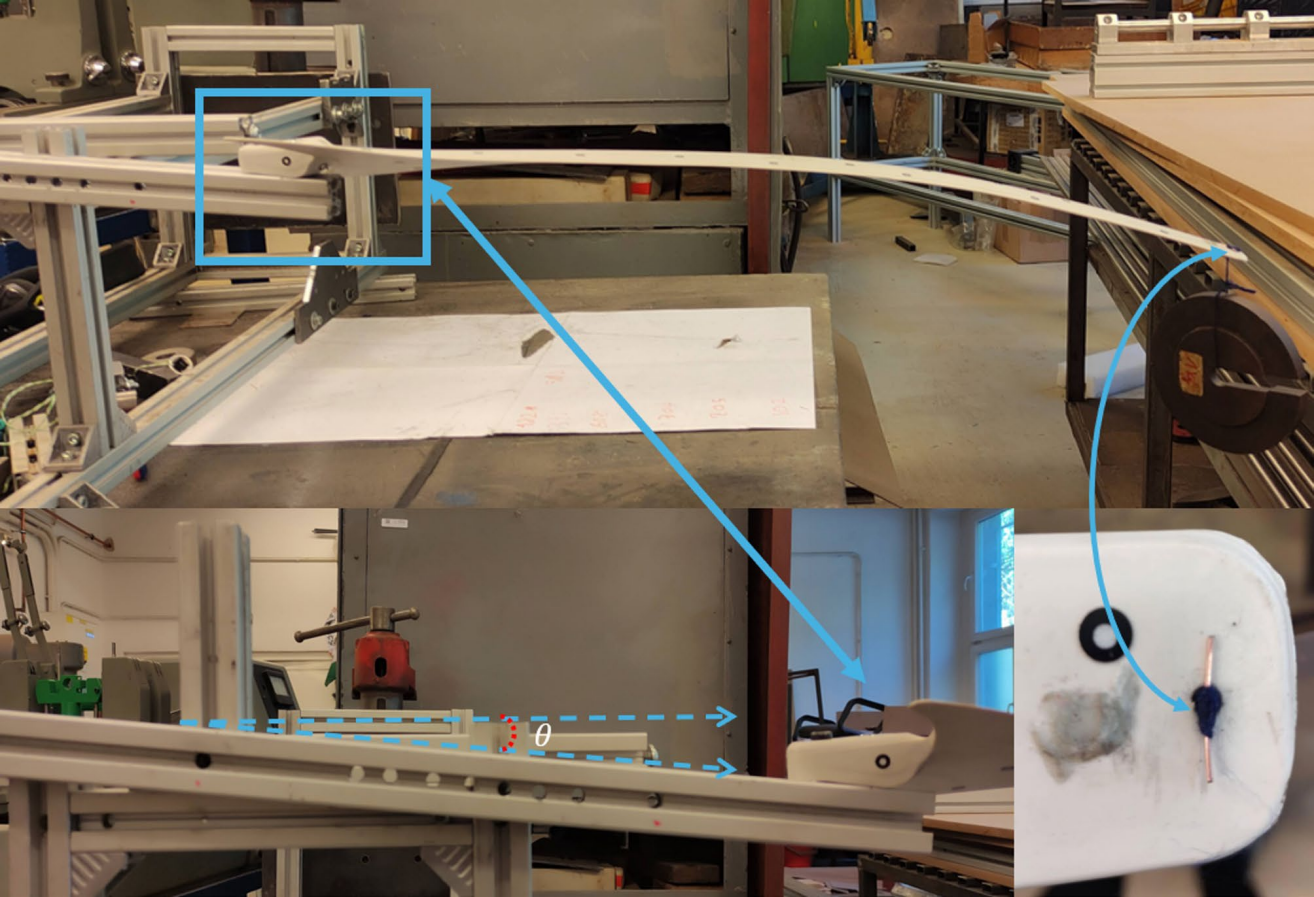
Mass release experimental set up, with $\:{\uptheta\:}=\:-5^\circ\:$.

#### Experimental data processing

The data processing starts with importing the acceleration data into MATLAB and then integrating the acceleration to obtain velocity and displacement using the cumulative trapezoidal numerical integration built-in function (cumtrapz). A drift appeared in the amplitude results which was removed by using the built-in function detrend, which removes the polynomial trend, as shown in Fig. 3.

**Fig. 4:**
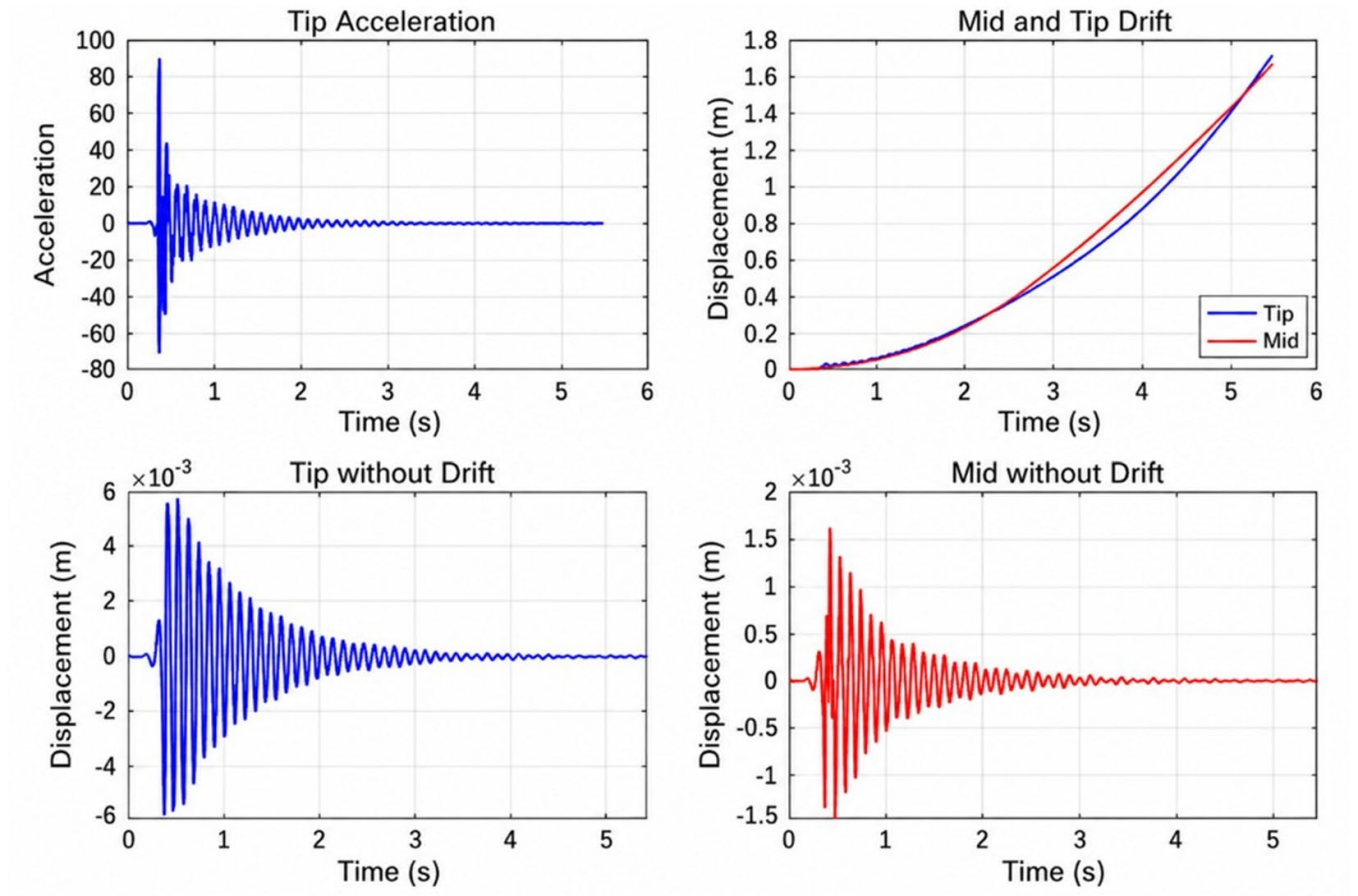
The drift effect due to double integration.

After extracting the amplitude from RT Pro 7.41 and preparing the data, the code then finds the highest ten absolute peaks from the integrated displacement and saves them into a matrix. The analysis of these matrices is divided into three groups where the displacement amplitude is used for comparison. Each group is normalized using the largest amplitude in its own set of points, which will be explained after discussing the groups.

The first group investigates the reduction of amplitude for the same dataset, where the first and largest peak is used to normalize the subsequent peaks. These values are directly related to the damping ratio (energy dissipation) or the logarithmic decrement of the blade.

The second group compares the amplitude under the effect of increasing mass for a specific angle. In this group, the largest amplitude, which occurs with the 1 kg mass, is used to normalize the peaks from other mass increments.

The third group compares the effect of blade angle under the same mass. This group uses the largest amplitude peak at the largest angle to normalize the other peaks. For example, when fixing the mass at 1 kg, the amplitude peak at 15° is used to normalize the peaks at the other angles under the same mass. This approach enables the investigation of ice-shedding effects under different angles and masses and is illustrated in Fig. 4.

**Fig. 5:**
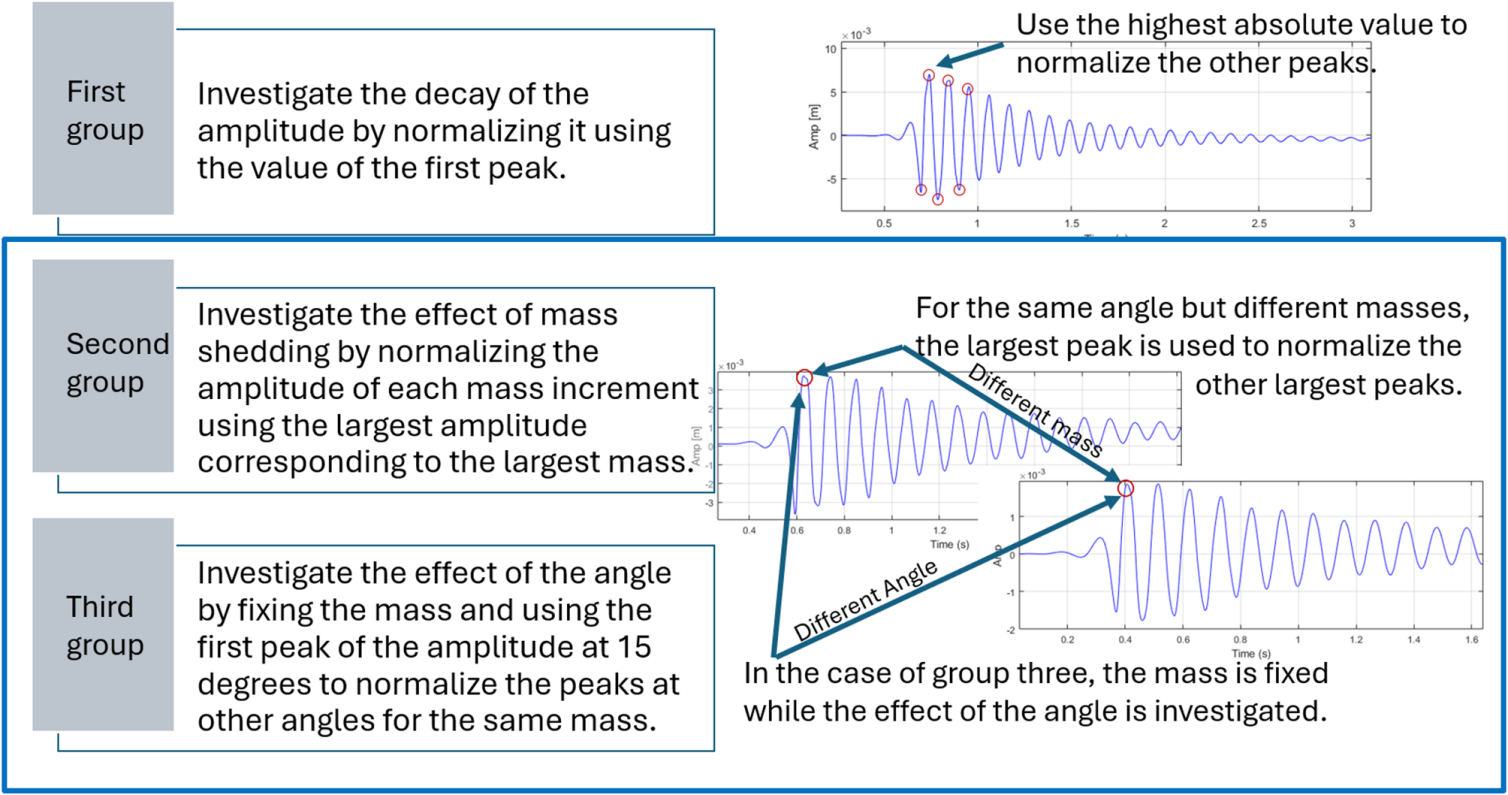
Illustrates the process of normalizing and analysing the data. The y-axis represents the amplitude, while the x-axis represents time.

## Finite element model

The aim of the random vibration analysis is to show the realistic wind excitation conditions under which the experimentally simulated ice-shedding event may occur in real-world applications. While the finite element model represents the baseline dynamic characteristics of the bare blade, the turbulent wind analysis provides the operational conditions that may lead to ice detachment during service. Once the shedding event occurs, the resulting transient vibration response is then investigated experimentally and numerically based on the validated structural dynamic behaviour of the blade.

The CAD model was created in two steps. The first step involved 3D scanning of the blade, while the second involved manual measurements to improve the accuracy of the scanned geometry. This model was then imported into ANSYS to determine the natural frequencies and compare them with the experimental results. Figure 5 illustrates the reverse engineering process of the blade.

Additionally, the density of the FEM model was adjusted to match the actual blade weight, while the Young’s modulus was chosen to minimize the error between the FEM and experimental frequencies using the root mean square error (RMSE, Eq. 1) for the first five natural frequencies.

1 $$\:\mathrm{RMS\:Error}=\sqrt{\frac{1}{N}\sum\:_{i=1}^{N}{\left(\frac{{f}_{i}^{\mathrm{FEM}}-{f}_{i}^{\mathrm{exp}}}{{f}_{i}^{\mathrm{exp}}}\right)}^{2}}\times\:100\mathrm{\%}$$

**Fig. 6:**
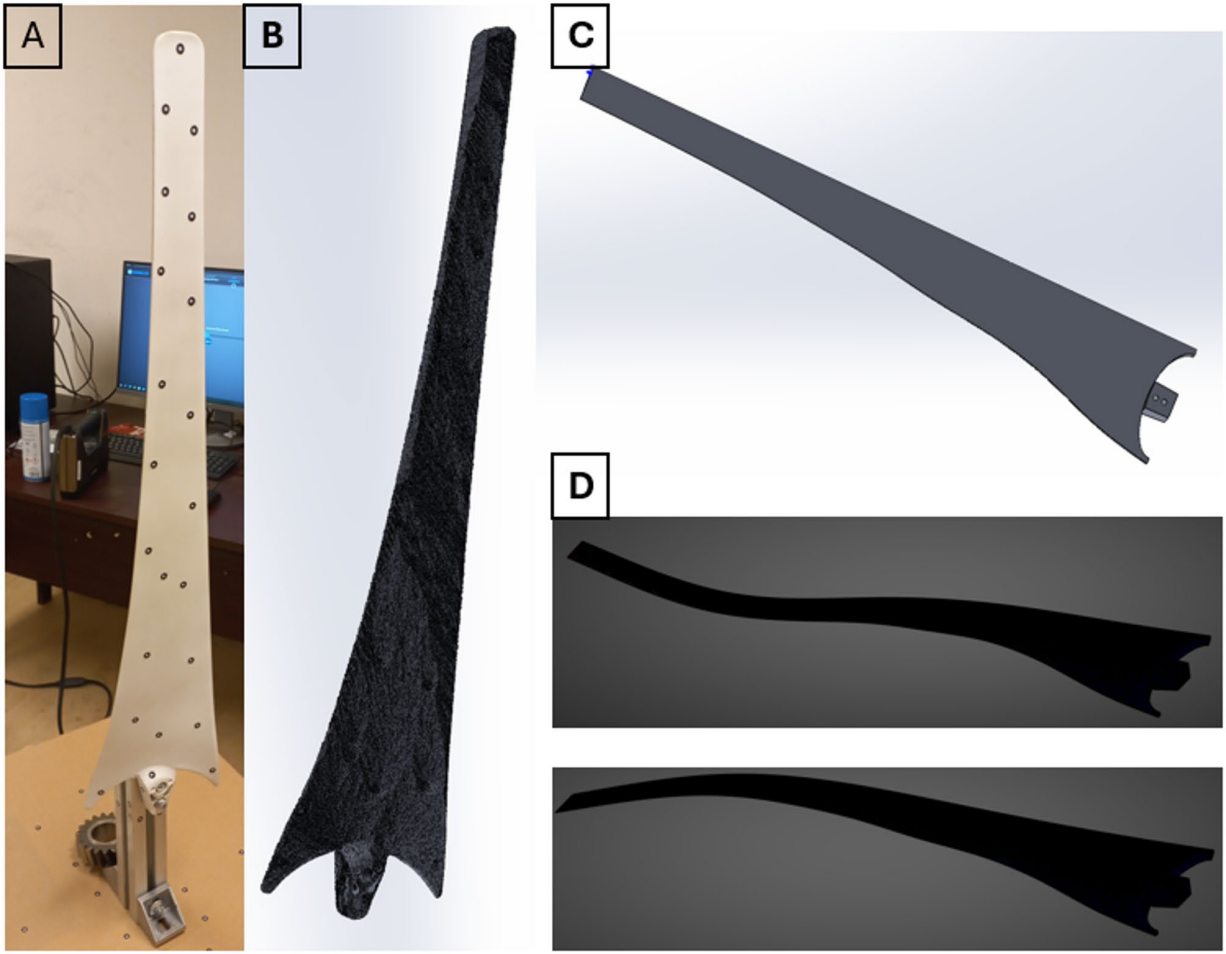
Reverse engineering of the CAD model. (**A**) 3D scanning, (**B**) initial 3D results, (**C**) finalized 3D model with manual measurements, (**D**) second and third mode shapes obtained from FEM.

Where $\:{f}_{i}^{\mathrm{exp}}$ is the experimental frequency of mode *\:i*, $\:{f}_{i}^{\mathrm{FEM}}$ is the FEM frequency of mode *\:i,* and *\:N* is the number of considered natural frequencies, which is five. The blade used in this study is a commercially available small-scale nylon wind turbine blade obtained from an online supplier^50^. The geometry was captured using 3D scanning and refined through manual measurements to ensure accuracy. This can be seen in Fig. 6.

The governing equation of motion for the FEM analysis is expressed as:

2 $$M~\ddot {u}\left( t \right)~+~C~\dot {u}\left( t \right)~+~K~u\left( t \right)~=~F\left( t \right)$$

where *u(t)* is the nodal displacement vector, *M* is the mass matrix, *C* is the damping matrix, *K* is the stiffness matrix, and *F(t)* is the external force vector.

For harmonic excitation, both the force and the displacement are assumed to have the time-harmonic form:

3 $$F\left( t \right)=~\hat {F}~{e^{i\Omega t}}$$

4 $$u\left( t \right)~=~\hat {u}~{e^{i\Omega t}}$$

where Ω is the angular frequency of excitation,$\:\:\widehat{F}\:=\:{F}_{max\:}{e}^{i\varphi\:}\:$is the complex amplitude of the applied load, and û is the complex displacement amplitude.

Differentiation gives:

5 $$\begin{gathered} \dot {u}\left( t \right)~=~i\Omega \hat {u}~{e^{i\Omega t}} \hfill \\ \ddot {u}\left( t \right)~=~ - {\Omega ^2}\hat {u}~{e^{i\Omega t}} \hfill \\ \end{gathered}$$

Substituting these expressions into (Eq. 2) and cancelling the common exponential factor leads to the harmonic equilibrium equation:

6 $$\left( { - {\Omega ^2}M~+~i\Omega ~C~+~K} \right)\hat {u}~=~\hat {F}$$

This equation describes the relationship between the complex force and displacement amplitudes. The physical time response can be obtained from the real part of û $\:e^{{\left( {i{{ \varOmega}}\:t} \right)}} .$.

The applied boundary conditions in the analysis are fixed at x = 0 and free at x = L. For an Euler–Bernoulli beam these conditions are:

- Fixed end at x = 0:

7 $$\begin{gathered} u\left( {0,~t} \right)=~0~ \hfill \\ \partial u/\partial x~\left( {0,~t} \right)~=~0 \hfill \\ \end{gathered}$$

(zero displacement and zero rotation)

- Free end at x = L:

8 $$\begin{gathered} EI~{\partial ^2}u/\partial {x^2}~\left( {L,~t} \right)~=~0 \hfill \\ \quad EI~{\partial ^3}u/\partial {x^3}~\left( {L,~t} \right)~=~0 \hfill \\ \end{gathered}$$

(zero bending moment and zero shear force)

where *\:E* is Young’s modulus, *\:I* is the second moment of area, and EI is the flexural rigidity.

### FE modal validation

The blade was modeled using solid linear tetrahedral elements with a manually defined element size of 1 mm. The mesh was generated using program-controlled meshing, which automatically determines element shape and connectivity. Similar finite element approaches using ANSYS have been widely applied in engineering optimization and structural analysis studies^51,52^. The mesh was generated using program-controlled meshing, which automatically determines element shape and connectivity. Larger element sizes (5 mm and above) could not be used due to the thin blade geometry and meshing limitations. The final mesh (1 mm) consisted of 1,552,984 nodes and 901,926 elements, providing sufficient resolution to capture both flap wise and chordwise bending behavior. The results of the mesh convergence study are presented in Table 1.

The mesh convergence results indicate that the selected discretization is sufficient and that further refinement is not required. Comparison with experimental measurements is presented in Sect. 5, where the FEM model is validated against the experimental data, confirming its ability to capture the bending-dominated dynamic behaviour of the blade.

**Table 1:** Mesh convergence analysis of the finite element model based on the first five natural frequencies.

Mode	1 mm Mesh (Hz)	3 mm Mesh (Hz)	Difference (Hz)	% Difference
1	7.9368	7.9449	0.0081	0.10%
2	25.636	25.691	0.055	0.21%
3	49.983	50.285	0.302	0.60%
4	63.199	63.393	0.194	0.31%
5	110.73	110.96	0.23	0.21%

Furthermore, the blade was fully constrained at the two root screw holes (Fig. 6) to replicate the experimental fixture.

### Random vibration analysis

Random vibration analysis provides a statistical view of the blade response under stochastic loading, but it does not capture all aspects of wind turbine blade behaviour^53^. In particular, it does not represent deterministic time histories, extreme gusts, or transient aerodynamic effects. Nonlinear aeroelastic effects, such as dynamic stall, load redistribution due to rotation, and control-system interactions, are not directly included. Also, random vibration analysis does not predict fatigue life or ultimate loads; it only gives RMS response quantities in the frequency domain. Therefore, this method is not meant to replace detailed aeroelastic time-domain simulations used for load prediction or certification.

Despite these limitations, random vibration analysis is still a useful tool for studying blade response under turbulent wind. Since atmospheric turbulence is stochastic and broadband, representing it in the frequency domain captures its main statistical characteristics. This method allows for quick evaluation of modal participation, identification of dominant frequency ranges, and estimation of RMS displacement, acceleration, or stress. It is particularly helpful for feasibility studies, comparing design options, and checking how the structure or material responds to turbulence. When used carefully, random vibration analysis gives a good understanding of blade vibration under realistic turbulent inflow.

In this study, random vibration analysis was used to evaluate the blade response under stationary stochastic loading. Unlike deterministic harmonic excitation, random loading is described by its power spectral density (PSD), showing how input energy is distributed across frequency. The analysis starts with the linear equation of motion of the finite element model.

Random vibration analysis was used to study the blade response under stationary stochastic loading. Unlike harmonic excitation, random loading is described by its power spectral density (PSD), which shows how the input energy is spread over frequency. The analysis starts from the linear equation of motion of the FEM model:

9 $$M~\ddot {u}\left( t \right)~+~C~\dot {u}\left( t \right)~+~K~u\left( t \right)~=~f\left( t \right)$$

where *M*, *C*, and *K* denote the mass, damping, and stiffness matrices, respectively, and *f(t)* is a zero-mean stationary random process. For stationary excitation, the response can be expressed in the frequency domain by applying the Fourier transform to (Eq. 9) which yields

10 $$\left( { - {\omega ^2}~M~+~i\omega ~C~+~K} \right)\hat {u}\left( \omega \right)~=\hat {f}~\left( \omega \right)$$

Here, $\:\hat{u}\left( {\omega \:} \right)$ and $\:\widehat{f}\left(\omega\:\right)$ are the complex frequency-domain displacement and force vectors. The random response is characterized statistically through its response PSD, which is related to the input $\:PSD\:{S}_{ff\left(\omega\:\right)}$ by

11 $$\:{S}_{uu}\left(\omega\:\right)\:=\:H\left(\omega\:\right){S}_{ff}\left(\omega\:\right){H}^{+}\left(\omega\:\right)$$

where $\:H\left( {\omega \:} \right)\: = \:( - \omega ^{2} \:M\: + \:i\omega \:\:C\: + \:K)^{{ - 1}}$ is the frequency response matrix and (·)⁺ denotes the Hermitian transpose. The root-mean-square (RMS) response is obtained by integrating the displacement PSD over the analysis bandwidth,

12 $$\:{u}_{rms}\:=\:\sqrt{{\int\:}_{0}^{\infty\:}{S}_{uu\left(\omega\:\right)d\omega\:}}$$

For practical implementation, a modal superposition approach is used. The displacement vector is expanded as u(t) = Φ q(t), where Φ contains the mass-normalized mode shapes. Substituting into (Eq. 9) and remultiplying by Φᵀ diagonalizes the equations, resulting in a set of uncoupled modal equations,

13 $$\:{\ddot{q}}_{r}\left(t\right)\:+\:2\:{\zeta\:}_{r}{\omega\:}_{r}{\dot{q}}_{r}\left(t\right)\:+\:{\omega\:}_{r}^{2}{q}_{r}\left(t\right)\:=\:{Q}_{r}\left(t\right)$$

where *r* denotes the mode number, $\:{\omega\:}_{r}\:$is the circular natural frequency, $\:{\zeta\:}_{r}$ is the modal damping ratio, and $\:{Q}_{r}\left(t\right)$ is the generalized force obtained by projecting the input onto the mode shape. The modal PSD of $\:{q}_{r}\:$is

14 $$\:{S}_{{q}_{r}{q}_{r}}\left(\omega\:\right)\:=\:{\left|{H}_{r\left(\omega\:\right)}\right|}^{2}{S}_{{Q}_{r}\:{Q}_{r}}\left(\omega\:\right)$$

with $\:{H}_{r}\left(\omega\:\right)$ the scalar modal frequency response function. The total response PSD is reconstructed by standard modal summation,

15 $$\:S_{{uu}} \left( {\omega \:} \right)\: = \:{{\varSigma}}\:_{r} \:S_{{q_{r} q_{r} }} \left( {\omega \:} \right){{\varPhi}}\:_{r} \:{{\varPhi}}\:_{{rT }}$$

ANSYS Mechanical handles this modal PSD method internally. The input excitation was defined as a one-sided PSD over the frequency range $\:[{f}_{min},\:\:{f}_{max}$] to include all relevant modes. Modal damping was set based on experimental values, and enough modes were included to ensure accurate modal participation up to the highest frequency of interest. The solution directly provides displacement, velocity, and acceleration PSDs along with their RMS values. This approach offers an efficient reliable way of predicting the probabilistic steady-state response of the structure under broadband excitation and is widely accepted in vibrational analysis of beams, blades, and lightweight structural components.

#### Von kármán turbulence power spectral density

The Von Kármán spectrum is commonly used to describe atmospheric turbulence^54^, as it gives a realistic representation of energy distribution over different spatial and temporal scales. For a single-point longitudinal velocity fluctuation, the one-sided PSD is:

16 $$\:{S}_{u}\left(\omega\:\right)\:=\:\frac{\left({\sigma\:}_{u}^{2}\:\frac{L}{\pi\:\:U}\right)}{{\left[1\:+\:{\left(1.339\:\frac{\omega\:L}{U}\right)}^{2}\right]}^{-\frac{5}{6}}}$$

and in ANSYS, the corresponding acceleration PSD is:

17 $$\:{S}_{a}\left(f\right)={\left(2\pi\:f\right)}^{2}{\phi\:}_{u}\left(f\right)\:[{m}^{2}/{s}^{2}/Hz]$$

where $\:{\sigma\:}_{u}^{2}$ is the variance of the turbulent velocity component, L is the integral length scale, U is the mean flow velocity, and ω is the circular frequency. This formulation captures both the inertial subrange and the low-frequency energy-containing range through a smooth roll-off governed by the integral scale. Compared with the Dryden and Kaimal spectra, the Von Kármán model provides a more physically consistent decay (roll-off) at high frequencies and is widely recommended for structural dynamics under wind loading.

In this study, the Von Kármán PSD was used to define the broadband wind-induced excitation for the random vibration analysis. The spectrum was discretized over the frequency interval of interest and supplied as the input PSD for the modal superposition procedure. This allows the resulting response PSD and RMS quantities to reflect the statistical characteristics of realistic atmospheric turbulence.

18 $$\:{\sigma\:}_{u}^{2}=\:{\int\:}_{fmin}^{fmax}{\phi\:}_{u}\left(f\right)\:df$$

The acceleration PSD input for the GOE-56 blade was generated over 0–100 Hz using 100 frequency points, clustered around the blade’s natural frequencies obtained by the FEM analysis and the modal experiment to ensure accurate resolution of resonance peaks. Furthermore, the dominant frequencies are in this range. The aerodynamic excitation was based on a Kaimal turbulence spectrum, and a drag coefficient of 0.02 was used to convert aerodynamic force PSD to acceleration PSD, accounting for the blade mass.

The acceleration PSD was applied in SI units (m²/s⁴/Hz) using the acceleration input option in ANSYS, rather than normalized g-units (g-force units), to maintain consistency with the physics-based wind excitation model. The input parameters used in this analysis are summarized in Table 2.

**Table 2:** Input parameters used for the random vibration analysis.

$\:\rho\:$ (air)	1.225 kg/$\:{m}^{3}$
Drag coefficient	0.3 for GOE-56
Mass (blade)	0.475 kg
Wind Speed	10 *\:m/s*
turbulence standard deviation	2 *\:m/s*
turbulence length scale	42 *\:m*
projected area	0.08 $\:{m}^{2}$

Figure 6 illustrates the points of Acceleration PSD vs. the frequency generated by using MATLAB. Then the values were implemented in ANSYS. Extra points around the natural frequency were generated to better capture the response of the blade around resonance. Furthermore, the logarithmic increment was used to calculate the damping ratio of the blade before implementing it in ANSYS. By using the amplitude data from the modal experiment, the damping ratio calculated from the logarithmic increment was 0.0447.

**Fig. 7:**
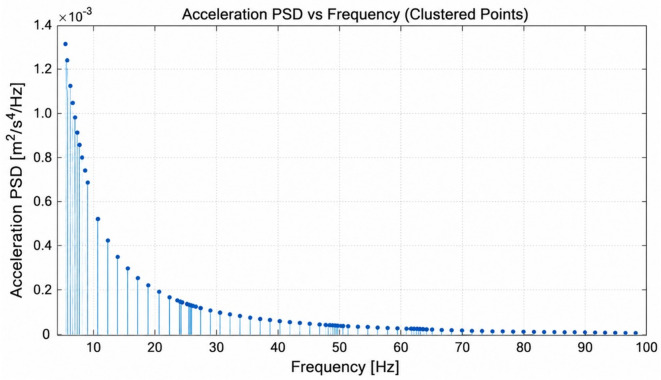
Acceleration PSD vs. Frequency generated points in MATLAB by using (Eq. 17) to find the PSD.

A mean wind velocity of 10 m/s was used, representative of typical small-scale wind conditions. While the resulting RMS tip displacement and acceleration are small due to the short, stiff blade, the analysis demonstrates the methodology for capturing stochastic dynamic response.

## Neural network

It is possible to make initial guesses (predictions) of the wind turbine vibration using Artificial Intelligence (AI), which is a powerful tool for prediction if sufficient data exists to train the AI model. In this research, the results obtained from the experiments were used as a dataset to train the neural network. The considered data points are the first five peaks of amplitude for each measured point. Hence, the total number of data points equals five times the number of measurement locations, which equals 350 points.

The input layer of the neural network consists of the sensor location, mass location, and mass value, which define the experimental configuration of the blade. The output of the network is the largest amplitude peak obtained after mass release. The first peak is selected as it represents the maximum dynamic response of the blade and corresponds to the instant where the highest stress and risk of structural failure are expected. Since subsequent peaks are governed mainly by damping effects and exhibit reduced amplitudes, the first peak provides the most critical and representative measure of the blade response under impulsive loading.

### Multi-layer perceptron (MLP)

Multi-Layer Perceptron (MLP) is a type of artificial neural network (ANN) used in deep learning, consisting of fully connected neurons with nonlinear activation functions. These neurons are organized in layers and can distinguish data that is non-linearly separable. Figure 7 illustrates the layer structure used in this study.

Equations (10–12) are used in the neural networks, where the results of these equations are criteria to help in improving the accuracy of the model. The equations include the determination coefficient $\:{(R}^{2}),$ the mean absolute error (MAE), and the root mean squared error (RMSE)^55^.

19 $$\:{R}^{2}=1-\left[\frac{\sum\:_{i=1}^{N}{(Q}_{{i}_{predicted}}-{Q}_{{i}_{observed}}{)}^{2}}{\sum\:_{i=1}^{N}{(Q}_{{i}_{ovserved}}-\stackrel{-}{{Q}_{{l}_{observed}}}{)}^{2}}\right]\:$$

20 $$\:MAE=\left(\frac{1}{N}\right)\sum\:_{i=1}^{N}{(Q}_{{i}_{observed}}-{Q}_{{i}_{predicted}})$$

21 $$\:RMSE=\:\sqrt{\left(\frac{1}{N}\right)\sum\:_{i=1}^{N}{(Q}_{{i}_{observed}}-{Q}_{{i}_{predicted}}{)}^{2}}$$

Here, *N* represents the total number of samples, and $\:{Q}_{{i}_{observed}}$ represents the actual observed max displacement values and $\:{Q}_{{i}_{predicted}}$ represents the predicted displacement values. Furthermore, three different training algorithms were used the Levenberg-Marquardt (trainlm) Bayesian Regularization (trainbr) and Scaled Conjugate Gradient Backpropagation (trainscg) explained in the following sections.

**Fig. 8:**
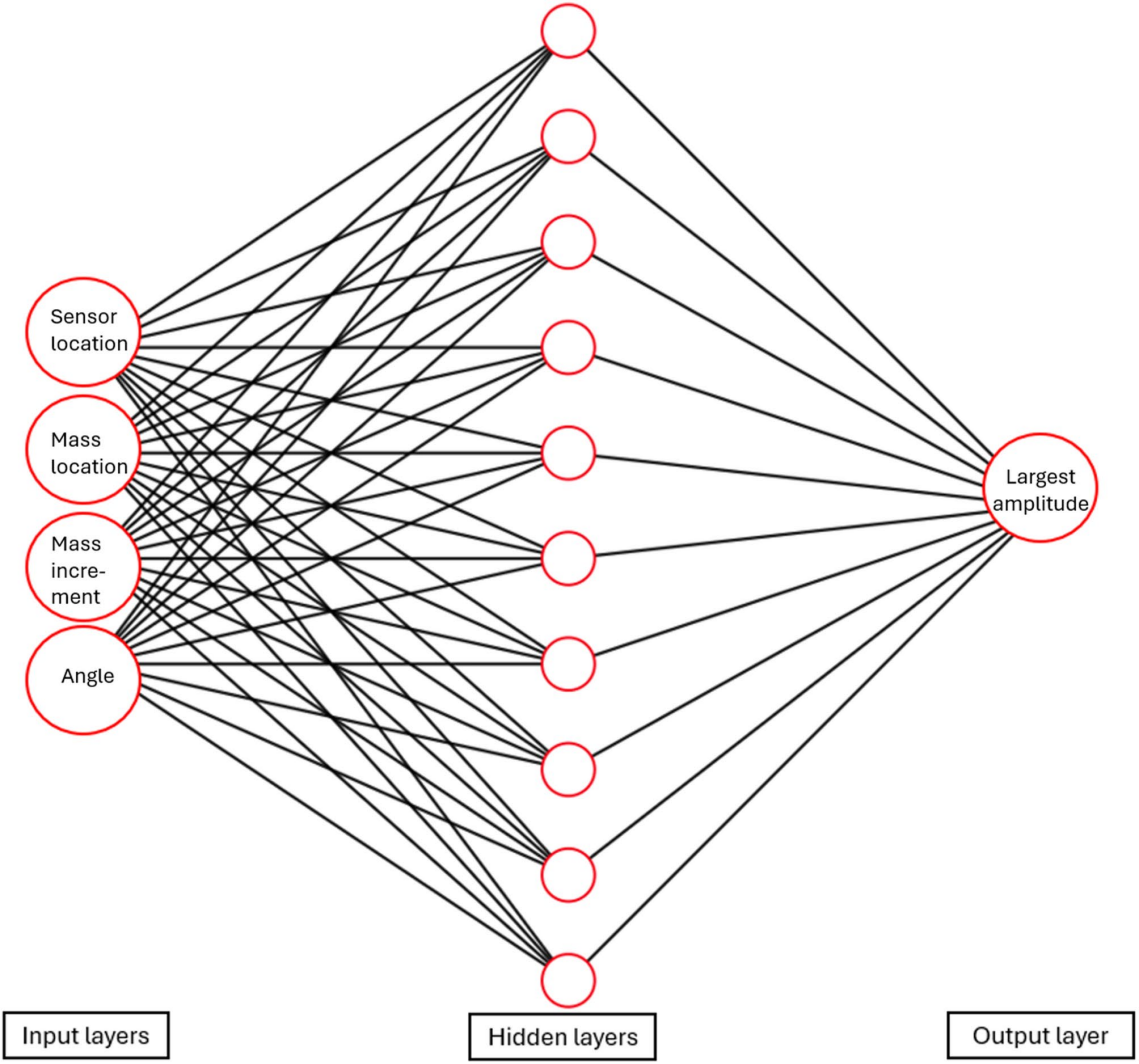
A schematic Diagram for the ANN and its relationship with the input and output parameters.

#### Scaled conjugate gradient (MLP_SCG)

The Scaled Conjugate Gradient (SCG) algorithm follows a training approach similar to other neural network models but is implemented within a multi-layer structure. The prediction process is based on several layers of interconnected units, where each unit is linked to the next layer through adjustable weights. During training, these weights are updated iteratively to minimize the prediction error^56^. The performance of the SCG algorithm can be further improved by applying different rescaling methods, such as standardized, normalized, and adjusted normalized scaling^57^.In this research, only the normalized scaling method was considered.

#### Levenberg–Marquardt algorithm (MLP_LMA)

The algorithm, first published by Kenneth Levenberg in 1944, has become widely used in neural network applications. The Levenberg–Marquardt Algorithm (LMA) is commonly used to train multi-layer perceptron (MLP) neural networks. This is achieved by iteratively identifying the best nonlinear function that fits a given dataset. The LMA combines the Gauss–Newton Algorithm (GNA) and the gradient descent method, allowing it to benefit from the advantages of both approaches. Compared with the GNA, the LMA is more robust, as it can converge even when the initial guess is not close to the optimal solution. However, the LMA is generally slower than other training algorithms when the objective function and the initial guess are reasonably accurate^58^.

#### Bayesian regularization (BRA)

Bayesian Regularization is a different training approach used for neural networks. In cases of small datasets or noisy data, this method often provides improved performance, at the expense of longer training time. This improvement is achieved by updating the network weights while incorporating prior knowledge, leading to a penalization of large weights and a more stable solution between the network layers^59^.

Figure 8 illustrates the flowchart of the neural networks. It shows the steps to process the inputs and how the layers are used and adjusted to train the network.

**Fig. 9:**
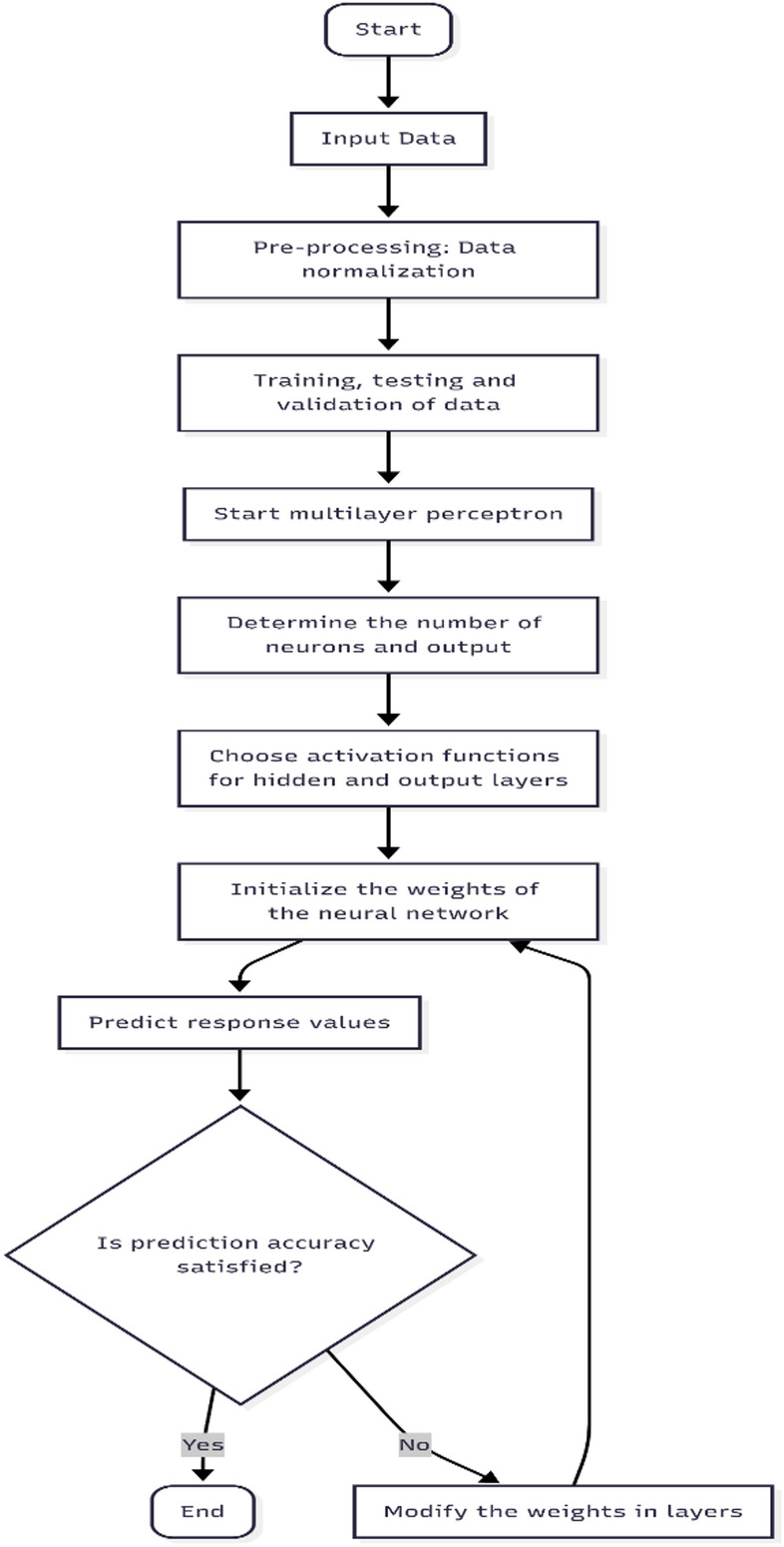
Flowchart for the process used to determine the prediction.

### Sensitivity analysis

A sensitivity analysis was performed using the trained neural network model to assess the relative influence of the input parameters which are sensor location, mass location, mass increment, and blade angle on the predicted vibration response. In this procedure, one input parameter was varied individually over its entire range while the remaining input parameters were kept fixed at their mean values. The sensitivity index for each input parameter was calculated based on the variation in the predicted output as follows:

$$\:{S}_{i}\:\:=\frac{\left({y}_{max}-\:{y}_{min}\right)}{{y}_{mean}}$$

where $\:{S}_{i}$ is the sensitivity index of the i-th input parameter, y_max and y_min represent the maximum and minimum predicted outputs resulting from the variation of that parameter, and $\:{y}_{mean}$ is the mean predicted output value. To facilitate comparison among the input variables, the obtained sensitivity values were normalized with respect to the maximum sensitivity value according to:

$$\:{S}_{i,norm}\:=\:\frac{{S}_{i}}{max\left({S}_{i}\right)}$$

This analysis provides additional insight into the physical significance of the selected input variables and strengthens the predictive capability of the proposed neural network model.

## Results and discussion

The results and discussion section will start by introducing and discussing the results from the experiment followed by the FEM model validation, Random vibration and finally, by the neural networks.

### Experimental results

As discussed before in the methodology, the experimental results are divided into groups to simplify the presentation of the results as well as keeping the analysis comprehensive enough. The first group compare the values of the absolute peaks obtained from the amplitude. Figure 9 compares for the picked peaks and amplitude at both mid and tip sensors for a 1 kg mass hung attached at the tip for the blade when it is tilted by 5 degrees. Furthermore, the initial disturbance can be explained by the mechanism explained in the methodology in which the mass is hung attached using a rope and then released using a lighter. Even though each experiment included sufficient waiting time, and repeated tests were performed when high noise occurred, the initial noise could not be fully eliminated.

**Fig. 10:**
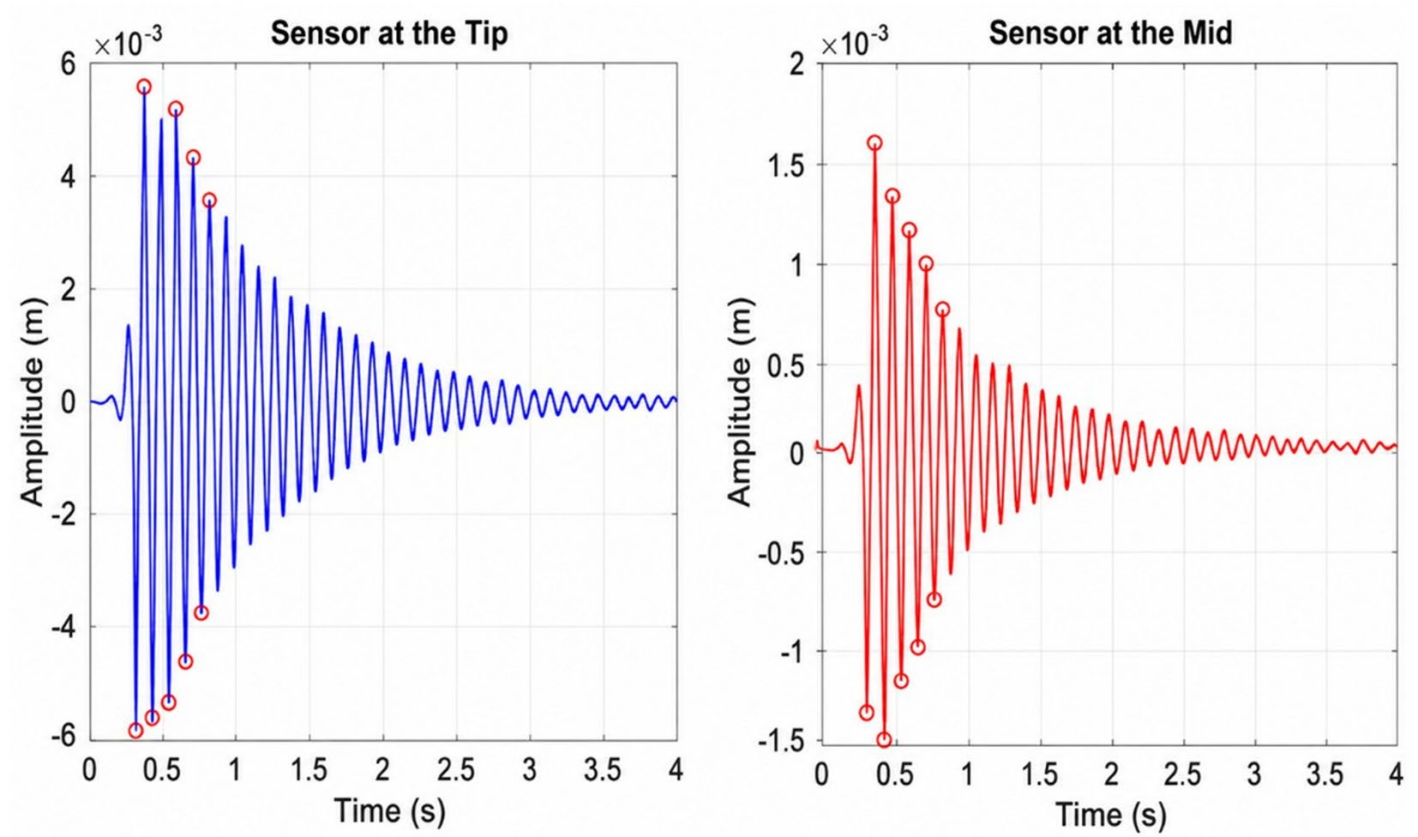
Illustration for the picked peaks and amplitude at both mid and tip sensors for a 1 kg mass hanged at the tip for the blade when it is tilted by 5 degrees.

Figure 10 illustrates the normalized amplitude values for different cases, where the x-axis represents the value of the attached mass and the y-axis represents the normalized amplitude values. The title of each plot refers to the attached mass location. The lines in the figures are included for better visualization. It is important to note that these values correspond to the absolute amplitude. Hence, the second point in every column corresponds to the first negative peak in the amplitude signal. It can also be observed that the rate of decay in the amplitude at the tip for the 0° angle is the largest, while the other cases show a slower decay. Additionally, the $\:0^\circ\:$ mid B plot shows the results of the mid-span sensor, while $\:0^\circ\:$ mid plot shows the tip sensor response. These plots show that the normalized response of the tip is similar to that at the mid-span.

**Fig. 11:**
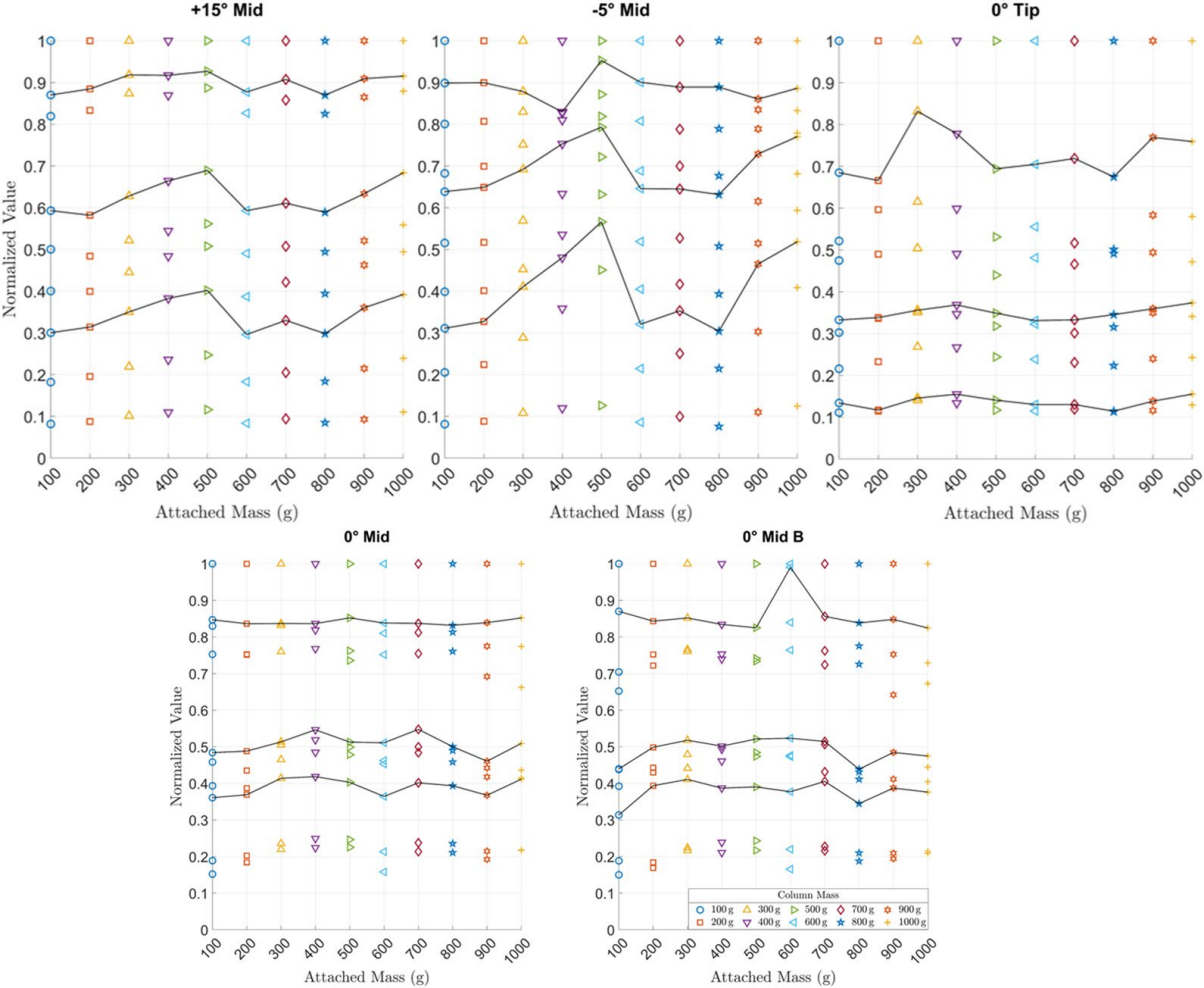
Normalization of Amplitude of the first group. $\:\mathrm{x}-$axis represents the value of the attached mass while the y-axis represents the normalized amplitude values. The title of each plot refers to the attached mass’s location. Lines in the figure are for better visualization.

Furthermore, the difference between the initial amplitude and the second peak increases with increasing the value of the attached mass. This shows the effect of pre-bending on the blade. The larger the pre-bending of the beam, the larger the stored potential energy and then released as kinetic energy and the system behaves as a free vibration under underdamped oscillation.

For an underdamped system^60^:

$$\:x\left( t \right)\: = \:x0\:\:e^{{ - \zeta \:\:\omega \:n\:t}} \:\:\left[ {cos\left( {\omega \:_{d} t} \right)\: + \:\left( {\zeta \:\:\frac{{\omega \:n}}{{\omega \:_{d} }}\:} \right)\:sin\left( {\omega \:_{d} t} \right)} \right]$$

where x₀ is the initial displacement, ζ the damping ratio, ωₙ the natural frequency, and $\:{\omega\:}_{d}$ the damped natural frequency. The ratio of successive peaks is governed by the logarithmic decrement:

$$\:\delta\:\:=\:ln\left(\frac{{x}_{1}}{{x}_{2}}\right)\:=\frac{\:2\pi\:\:\zeta\:}{\sqrt{1\:-\:{\zeta\:}^{2}}}\:\:$$

This can be further explained using Fig. 11, which illustrates the RMS values for the different normalized cases corresponding to the data in the second group, comparing the values obtained from the tip and mid-span sensors. The RMS values represent the kinematic (vibrational) energy calculated over the first ten captured peaks. It can be clearly observed that the response at the blade tip is consistently higher than at the mid-span for all tested cases. This consistent trend indicates that the response is primarily governed by the structural characteristics of the blade rather than by the specific excitation conditions of each case.

The higher response observed at the blade tip can be explained by the bending mode shapes of the blade. Since the blade behaves like a cantilever structure, the dominant bending modes produce increasing displacement toward the free end. Consequently, the blade tip is located closer to the anti-nodes of the mode shapes, while the mid-span lies in regions with lower modal participation. As a result, the impulsive excitation caused by ice release produces larger deflections and higher vibrational energy at the tip compared to the mid-span. The agreement between the measured responses and the expected modal behaviour confirms that the blade dynamics during ice shedding are dominated by bending effects.

In addition, the blade–rotor angular position has a clear influence on the RMS responses. Differences are observed among the tested angular positions (15°, 5°, 0°, and − 5°) for both tip and mid-span measurements. Since these angles represent the blade orientation relative to the rotor rather than the aerodynamic angle of attack, the variations in response might be due to changes in the gravitational and inertial load components acting along the blade during ice release. The consistent influence of blade orientation across different ice masses emphasizes its role in governing the transient response of the blade.

**Fig. 12:**
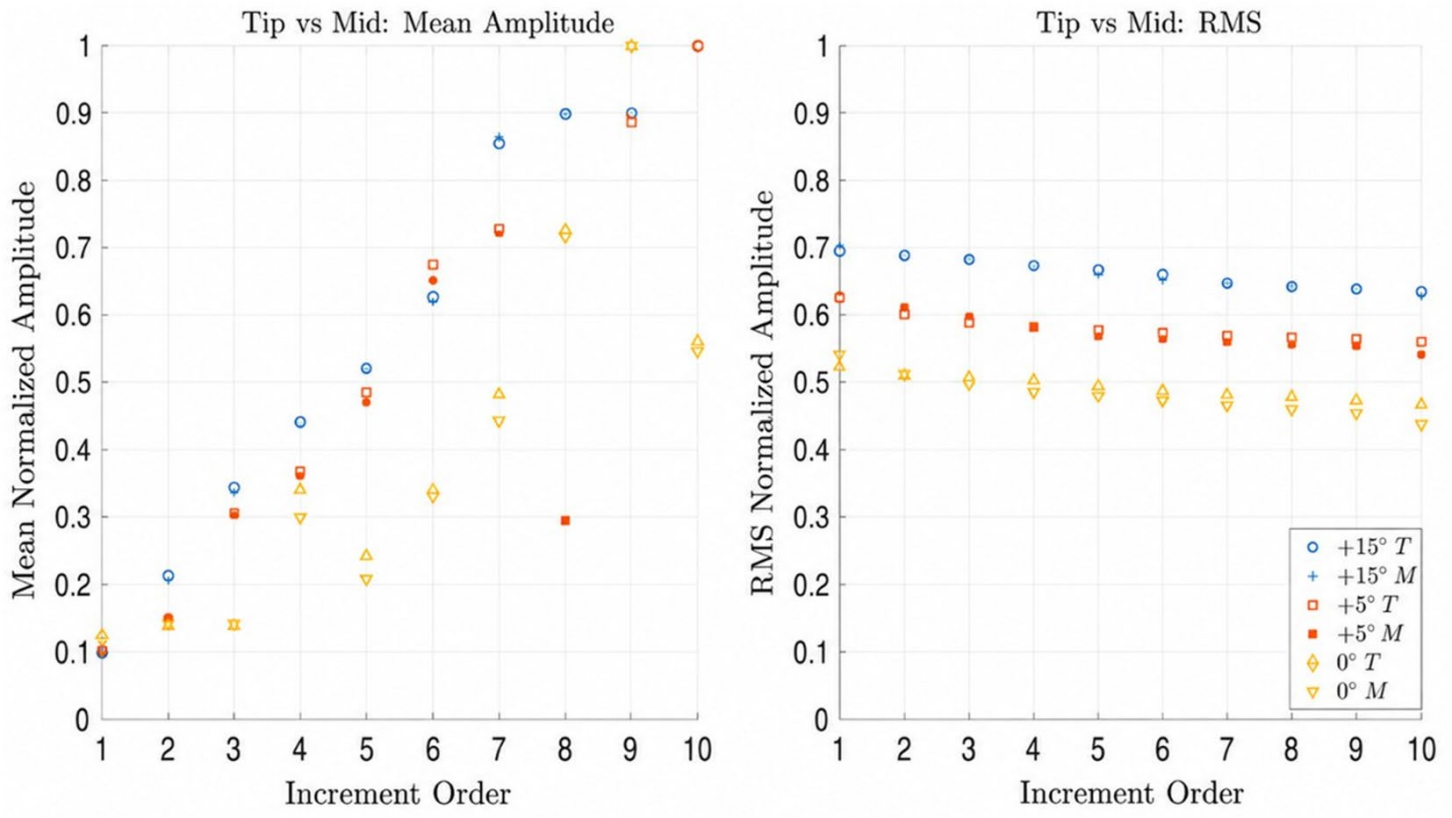
Mean and RMS values for Sensors attached at the Tip and Mid locations of the blade.

The final group of this analysis investigates the effect of blade angle for a specific mass. This is done by extracting the three largest peaks from each experiment and grouping them accordingly. The data is then normalized by dividing the amplitude of the largest peak by the others. For example, if the 15° amplitude at a mass of 500 g is the largest, it is used to normalize the amplitudes of the other angles at 500 g. This process is illustrated in Fig. 12 for the first three peaks. The figure is structured like a chart, where the x-axis is divided into ten sections corresponding to ten mass increments, and in each section, the normalized values are plotted vertically, with the first vertical value representing the first peak followed by the second and third peaks comparison. The titles of the sections in the figure show the location of the sensors.

**Fig. 13:**
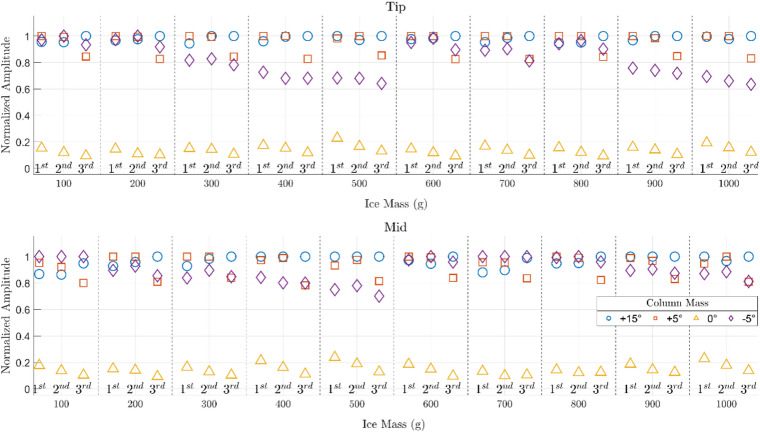
A comparison between first three peaks of amplitude for the same mass. The plot presents data obtained by the sensors at the tip and mid-span locations.

The analysis of normalized amplitude values indicates that the blade tip consistently shows a higher sensitivity to response decay than the mid-span across all ice masses and blade orientations. This expected behaviour supports the reliability of the experiments. The difference between the normalized values is larger at the tip, which is consistent with the expected behaviour of a cantilevered structure. As the ice mass increases from 100 g to 1000 g, the normalized amplitudes remain largely stable, suggesting that the largest peak dominates the response with these masses. Blade orientation also has a clear effect: the largest amplitudes are observed at + 15°, the lowest at 0°, and the intermediate angles (+ 5° and − 5°) fall between these extremes in most cases.

The triangular markers, representing the normalized amplitude at 0°, remain around 0.2 or lower across all experiments. This indicates that the blade experiences relatively low vibration in the horizontal configuration. This behaviour might be due to one or more of the following factors: constraints imposed by the root fixture (bolt-screw mechanism), the horizontal orientation reducing the gravitational component along the blade, or the ice-shedding impulses being less effective in exciting the dominant bending modes.

The noticeable jump in blade response observed at 600 g and higher mass values of attached mass can be attributed to two related factors. First, as the attached mass approaches and slightly exceeds the mass of the blade, the additional inertia significantly alters the modal participation, particularly for higher bending modes. Second, this behaviour reflects a dominant shift in modal contribution, where the relative influence of different modes on the overall dynamic response changes as system parameters are modified. This combination leads to the sudden increase in normalized amplitude observed in the experiments, consistent with established nonlinear vibration theory for multi-degree-of-freedom systems^61^. Such behaviour underscores the importance of considering mass ratios and modal interactions when analysing transient dynamic responses in wind turbine blade^61^.

Repeated measurements cluster closely at both tip and mid-span, confirming the reliability and repeatability of the experiments. Slight scatter is observed at the mid-span for intermediate angles. Overall, these observations confirm that the blade response is governed by its structural dynamics, with the tip response amplified by modal anti-nodes and the overall behaviour influenced by both ice mass and the gravitational and inertial effects associated with blade orientation.

### FE model validation

Since the low-order natural frequencies dominate the structural response^62^ and are also the most accurately measured in the experiments^63^, the first five natural frequencies were used to validate the Finite Element Model. However, challenges remain in the validation due to uncertainties in the Young’s Modulus of the glass-filled nylon blade. Therefore, the validation was performed using the root mean square percentage error. Additionally, the weight and density of the blade were directly measured to support the model accuracy.

Table 3 presents the RMS for different densities and *\:E* values which is calculated using (Eq. 1). The density of the blade is $\: \approx \:1400\:kg/m^{3}$ calculated by dividing the weight over the volume. However different densities are shown in the table just for comparison. The first five experimental natural frequencies are {8.8, 25.07, 44.9, 64.19, 117} *\:Hz*. The table just shows a sample of the simulation data. The RMS increases with decreasing Young’s modulus .

**Table 3:** The effect of density and Young’s modulus variation on the natural frequencies compared to the measurement.

ρ (kg/m³)	Young’s Modulus E (GPa)	f₁ (Hz)	f₂ (Hz)	f₃ (Hz)	f₄ (Hz)	f₅ (Hz)	RMS Error (%)
1400	5.35	7.9368	25.636	49.983	63.199	110.73	7.22
1400	5.20	7.8247	25.274	49.277	62.307	109.16	7.38
1400	5.66	8.1635	26.368	51.410	65.005	113.89	7.72
1500	5.00	7.4126	23.943	46.682	59.026	103.41	9.84
1150	2.50	5.9862	19.335	37.699	47.667	83.514	25.63
1400	3.00	5.9433	19.197	37.429	47.326	82.915	26.15
1400	2.19	5.0779	16.402	31.979	40.435	70.843	36.71

The combination $\:\rho \: = 1400\:kg/m^{3} ,\:E = 5.35\:GPa$ provides the best overall agreement with experimental transverse modes.

### Random vibration

The random vibration analysis provides RMS estimates of displacement, acceleration, and stress, which are used to assess the statistical dynamic response of the blade under turbulent wind excitation. The results are interpreted as indicators of vibration severity and modal sensitivity rather than as the exact deterministic load predictions.

Reporting the RMS deformation, acceleration, and normal stress in the X, Y, and Z directions provides a comprehensive view of the blade’s dynamic response under stochastic wind excitation. In this study, the wind is aligned with the X-axis, producing a zero angle of attack. The X-direction captures chordwise bending along the wind, the Y-direction captures flap wise bending perpendicular to the wind, and the Z-direction captures torsion or out-of-plane motion. Analysing all three components ensures that the dominant modes and critical locations for displacement, acceleration, and stress are identified. This multidirectional assessment is particularly important for lightweight blades, where coupling between bending and torsion can affect structural performance, fatigue, and serviceability. Figure 13 shows the first, third and fifth mode shapes, deformation and acceleration at different directions while Table 2 presents the range (Red is maximum while blue is minimum).

These values are related to Fig. 6 that shows the wind modelling values, where the stochastic wind acting on the blade was modelled using the Kaimal turbulence spectrum, which defines the power spectral density (PSD) of wind speed fluctuations as a function of frequency. The fluctuating aerodynamic force on the blade was calculated using a linearized drag formulation, $\:F\left(t\right)\approx\:\rho\:{C}_{d}AU u\left(t\right)$, Low-frequency components of the turbulent wind carry the largest energy and primarily excite bending modes of the blade, while high-frequency components contain less energy and excite higher-order modes. To accurately capture the dynamic response, the input PSD was therefore clustered around the natural frequencies corresponding to the dominant modes. The resulting acceleration PSD was applied in ANSYS random vibration analysis, yielding RMS displacement, acceleration, and stress values that statistically describe the blade’s response under turbulent wind excitation.

**Fig. 14:**
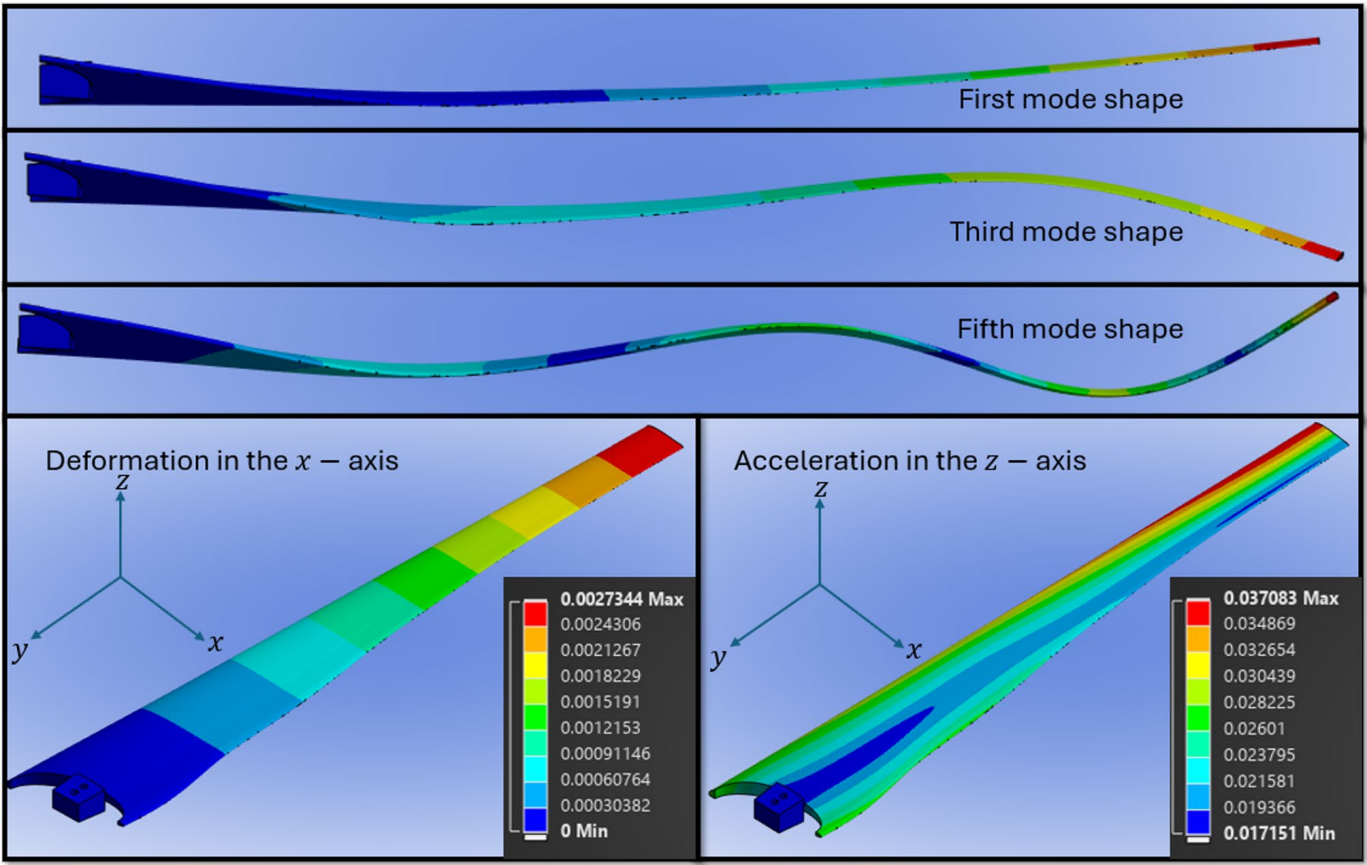
First, third and fifth mode shapes as well as deformation in the x direction and acceleration in the z- direction obtained by ANSYS workbench. Red is maximum and blue is minimum where Table 2 shows the range.

**Table 4:** Extremes of Deformation, Acceleration, and Stress.

Deformation [$\:{10}^{-7}\:m$]	x	y	z
	(0, 4.1)	(0, 5.1)	(0, 0.32)
Acceleration [$\:m/{s}^{2}]$	(0, 0.034)	(0, 0.028)	(0, 0.0024)
Stress $\:\left[Pa\right]$	(0, 1593)	(0, 3146)	(0, 3146)

Figure 14 illustrates how the acceleration PSD changes with frequency. The figure shows the dynamic response of a blade at zero angle of attack where the acceleration is applied along the chord axis. Furthermore, the response remains smooth around the first natural frequency reflecting the limited energy in the turbulent wind spectrum at this frequency and the directional alignment of the excitation. Higher-frequency modes show more pronounced contributions due to stronger modal participation and coupling in the frequency domain as well as the higher energy of the turbulent spectrum (Table 4).

**Fig. 15:**
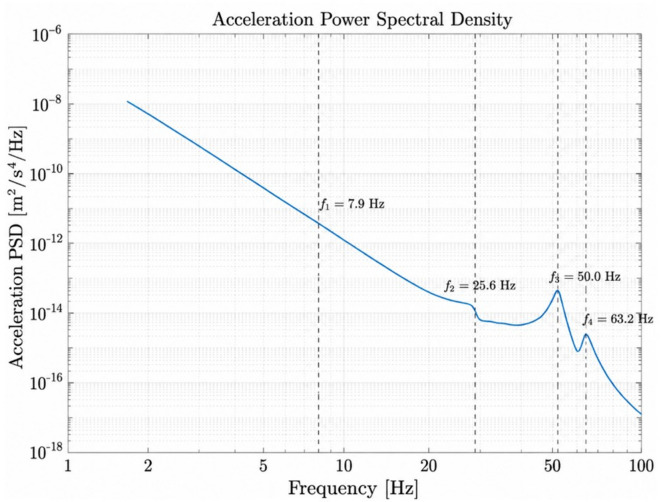
Acceleration Power Spectral Density vs. Frequency showing the reaction of the blade and resonance at the first four natural frequencies.

### Neural networks

The MATLAB multilayer perceptron (MLP) neural network was trained using three algorithms: Levenberg–Marquardt (LM), Scaled Conjugate Gradient (SCG), and Bayesian Regularization (BR), on a total of 1*4*0 samples. The input set includes the ten mass increments, their corresponding angles, and binary inputs (0 and 1) indicating the mass and sensor locations, resulting in 1*4*0 input samples. The dataset was randomly divided into 70% for training, 15% for validation, and 15% for testing, corresponding to 98, 21, and 21 samples, respectively.

#### MATLAB results and discussion

Figure 15 illustrates the neural network model implemented in MATLAB by comparing the predicted outputs with the measured samples. The results indicate that the Bayesian Regularization algorithm provides the closest agreement between predicted and measured values, while the SCG algorithm shows the weakest performance, with correlation coefficients of *R* = 0.97 and *R* = 0.94, respectively. The LM algorithm achieves an intermediate performance with *R* = 0.96. The correlation coefficient R quantifies the linear relationship between predicted and measured values, where *R* = 1 represents perfect correlation and *R* = 0 indicates no correlation. The diagonal line corresponds to the ideal *R* = 1 condition.

**Fig. 16:**
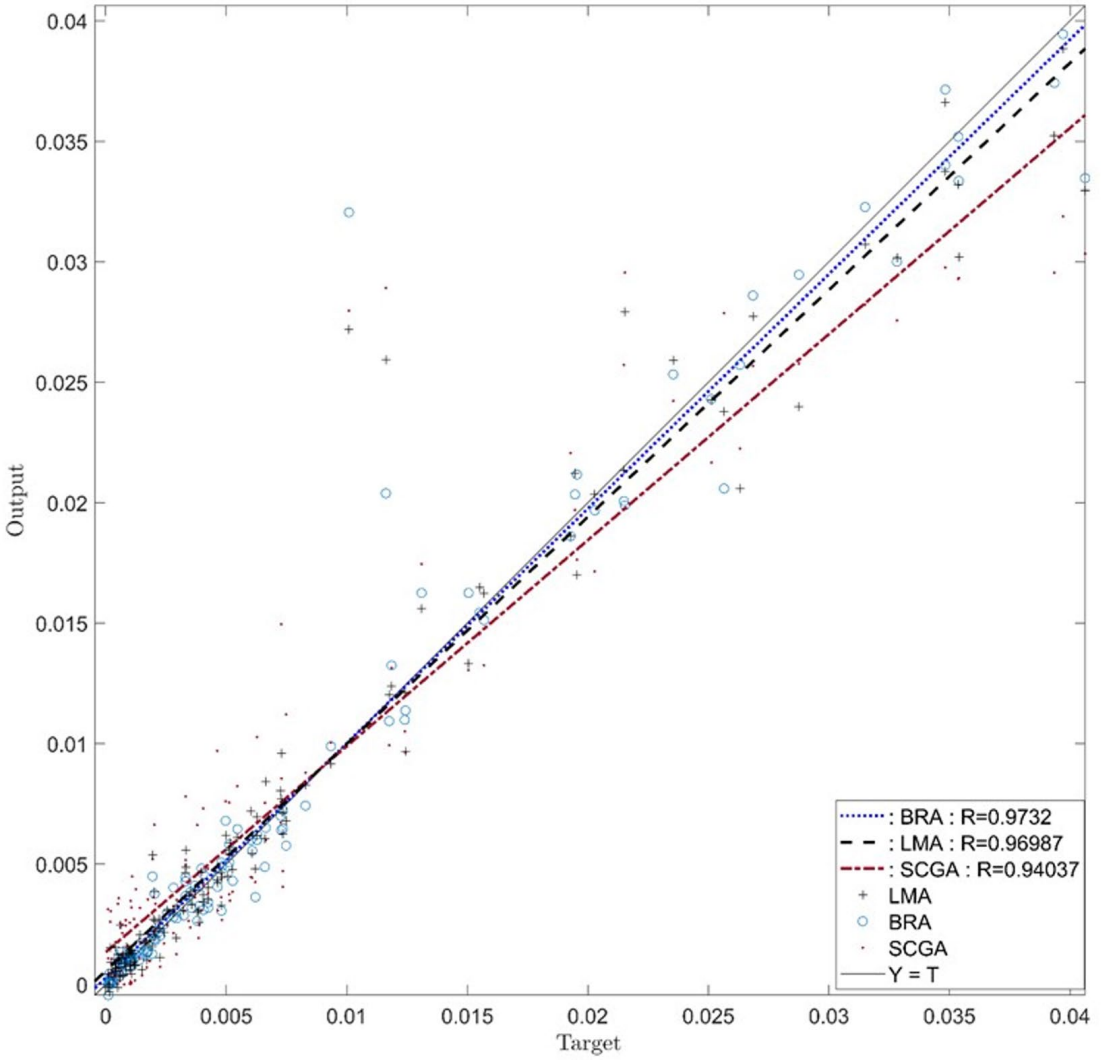
Schematic diagram for MATLAB neural network and the regression of the three different algorithms.

**Fig. 17:**
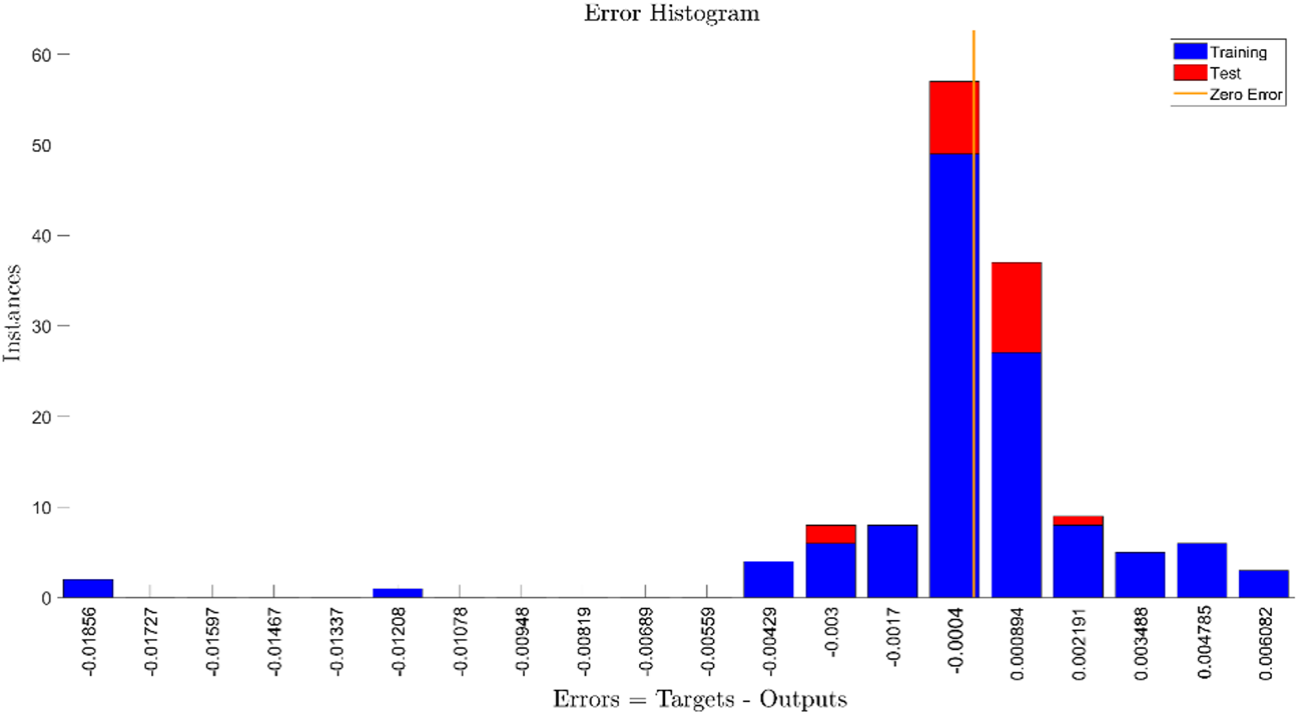
A statical distribution for the errors of Bayesian regularization, the algorithm with optimum performance) (MAE).

Figures 16 and 17 present the performance of the best-performing training algorithm (Bayesian Regularization), which achieved a minimum performance value of 1.4856 × 10⁻⁶ at epoch 168. Figure 16 shows the error evolution using the mean absolute error (MAE), while Fig. 17 presents the corresponding mean squared error (MSE). These results demonstrate that the neural network is capable of capturing the underlying trends in the experimental data within the limits of the available dataset

**Fig. 18:**
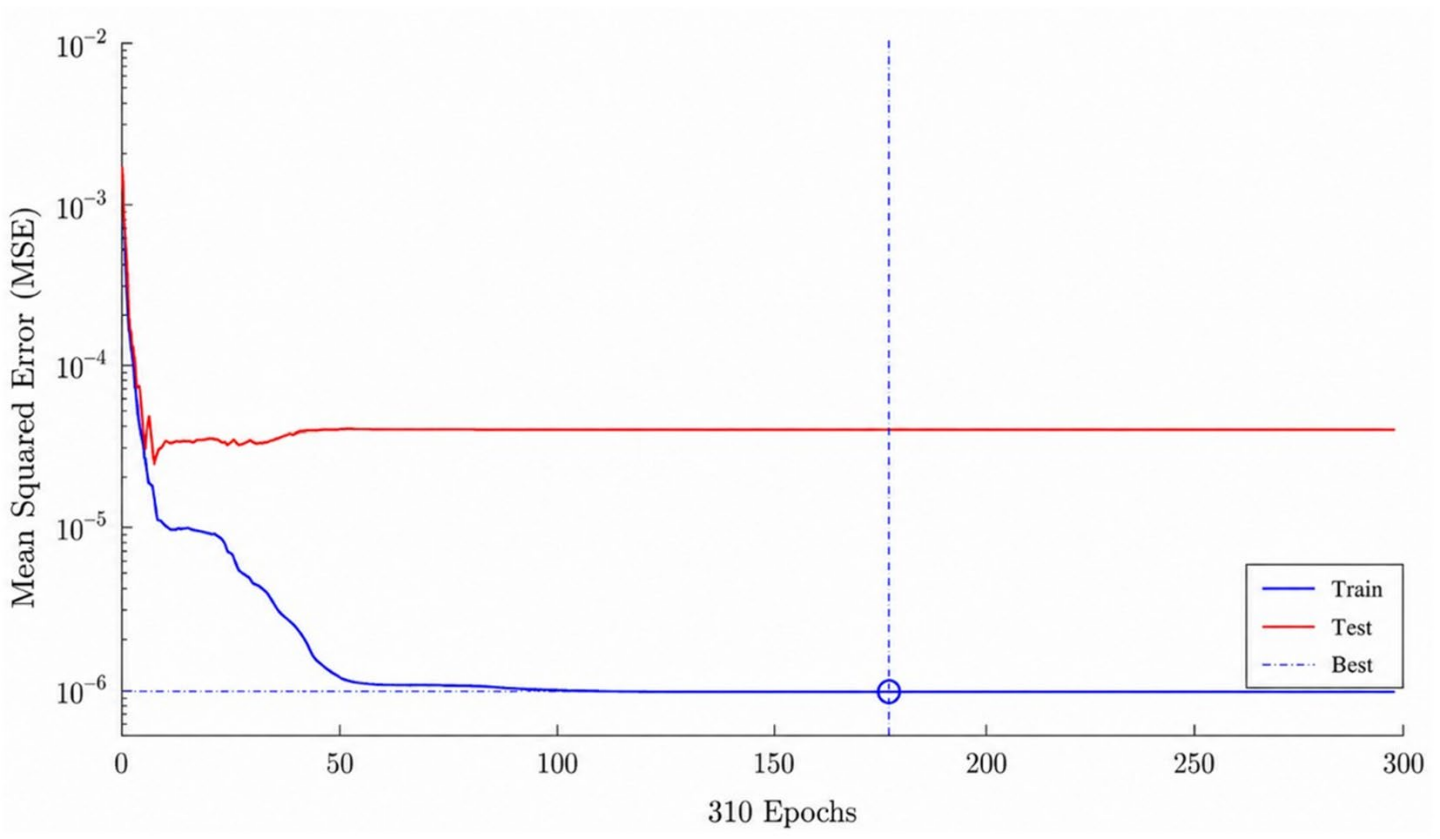
The means square error for the training, validation and test samples (MSE).

An epoch refers to one complete forward and backward pass of the entire training dataset through the network during the training process. To further assess the predictive capability of the developed neural network model, its performance was compared with that of a conventional linear regression model, which adopted a baseline predictive approach for relatively small datasets due to its simplicity, low computational cost, and reduced risk of overfitting (Fig. 18). It is particularly suitable when the relationship between the input parameters and the output response is approximately linear. To justify the use of the neural network model, a linear regression model was developed in MATLAB using the same input dataset and compared with the selected neural network training algorithms. The prediction performance of each model was evaluated using the root mean square error (RMSE), calculated as:

The obtained RMSE values were 0.0060 for the linear regression model, 0.0042 for the scaled conjugate gradient neural network, and 0.0032 for the Bayesian regularization neural network. The lower RMSE values achieved by the neural network models confirm their superior capability in capturing the nonlinear interaction among the input parameters, resulting in improved prediction accuracy compared with linear regression.

The results suggest that, with an expanded dataset, neural network-based approaches could provide a useful tool for supporting the analysis of ice-related vibration behaviour, rather than serving as a standalone predictive model.

## Conclusion

The present study established a repeatable and controlled experimental framework for investigating the transient vibration response of a small horizontal-axis wind turbine blade under simulated ice-shedding conditions. The experimental study used a controlled mass-release methodology which allows a systematic investigation of the transient response under different released masses and blade orientation angles. The results showed that the structural response is primarily governed by the first peak amplitude. Increasing the released mass from 100 g to 1 kg resulted in a clear increase in overall blade deflection, while blade orientation significantly influenced the transient response due to the combined effects of gravity and inertia. A noticeable change in the vibration response is observed when the released mass becomes comparable to the blade mass or higher, demonstrating the sensitivity of the proposed experimental methodology to variations in mass loading.

The normalized amplitude analysis further confirmed the dominant role of the first response peak, with positive blade inclinations leading to amplified tip deflections, whereas the lowest amplitudes were observed at 0°. In addition, the close results of repeated measurements across all test cases confirm the reliability and repeatability of the proposed experimental methodology.

The finite element model provided a consistent numerical representation of the blade behavior. Mesh convergence analysis showed that the selected discretization is sufficient, with frequency differences below 0.6%, and validation against experimental measurements confirms that the model accurately captures the dynamics of the blade.

The random vibration analysis based on the Von Kármán and Kaimal turbulence spectra was used to investigate the dynamic behavior of the blade under stochastic wind excitation. The response spectra showed clear resonance peaks at the natural frequencies, indicating that the blade behavior is governed by its modal characteristics. This provides insight into how turbulent wind can excite specific vibration modes and potentially contribute to conditions leading to ice-shedding events.

Neural network models trained on experimental data successfully captured trends in peak displacement amplitude as a function of released mass, sensor location, and blade orientation. A comparative analysis with linear regression confirmed the superior performance of the neural network approach, where Bayesian Regularization achieved the lowest RMSE value of 0.0032 compared with 0.0042 for SCG and 0.0060 for linear regression. These results demonstrate that data-driven approaches can support trend analysis and predictive assessment of transient ice-shedding behavior within the limits of the available dataset.

Overall, the main contribution of this study lies in the development of a reproducible experimental methodology for simulating ice-shedding events and analyzing the resulting transient blade response. The combined use of experimental testing, finite element modelling, random vibration analysis, and data-driven approaches provides a consistent framework for interpreting both transient and resonance-driven behavior. Future work should focus on improving the release mechanism to reduce initial noise and enhance experimental repeatability, expanding the dataset to improve neural network performance, studying extended numerical analysis for more complex loading conditions.

## Data Availability

The datasets used and/or analysed during the current study available from the corresponding author on reasonable request. Iyad F. Al-Najjar, E-mail address: *IyadF.AL-Najjar@gmail.com*. The data obtained and analysed during the current study, including experimental measurements, finite element model results, and blade geometry information, are available from the corresponding author on reasonable request.
